# A new peptide inhibitor of C1QBP exhibits potent anti‐tumour activity against triple negative breast cancer by impairing mitochondrial function and suppressing homologous recombination repair

**DOI:** 10.1002/ctm2.70162

**Published:** 2025-01-02

**Authors:** Xingxing Li, Yue Wu, Min Zhang, Fengliang Wang, Hong Yin, Yanrong Zhang, Shuli Zhao, Jiehua Ma, Mingming Lv, Cheng Lu

**Affiliations:** ^1^ Department of Breast Women's Hospital of Nanjing Medical University Nanjing Women and Children's Healthcare Hospital Nanjing China; ^2^ The State Key Laboratory of Pharmaceutical Biotechnology Division of Immunology Medical School Nanjing University Nanjing China; ^3^ Nanjing Women and Children's Healthcare Institute Women's Hospital of Nanjing Medical University Nanjing Women and Children's Healthcare Hospital Nanjing China; ^4^ General Clinical Research Center Nanjing First Hospital Nanjing Medical University Nanjing China

**Keywords:** anti‐tumour peptide, C1QBP, homologous recombination repair, mitochondrial function, targeted protein degradation

## Abstract

C1QBP exhibits heightened expression across a spectrum of tumours, thereby fostering their proliferation and metastasis, rendering it a pivotal therapeutic target. Nevertheless, to date, no pharmacological agents capable of directly targeting and inducing the degradation of C1QBP have been identified. In this study, we have unveiled a new peptide, PDBAG1, derived from the precursor protein GPD1, employing a peptidomics‐based drug screening strategy. PDBAG1 has demonstrated substantial efficacy in suppressing triple‐negative breast cancer (TNBC) both in vitro and in vivo. Its mechanism of action involves mitochondrial impairment and the inhibition of oxidative phosphorylation (OXPHOS), achieved through direct binding to C1QBP, thereby promoting its ubiquitin‐dependent degradation. Concomitantly, due to metabolic adaptability, we have observed an up‐regulation of glycolysis to compensate for OXPHOS inhibition. We observed an aberrant phenomenon wherein the hypoxia signalling pathway in tumour cells exhibited significant activation under normoxic conditions following PDBAG1 treatment. Through size‐exclusion chromatography (SEC) and isothermal titration calorimetry (ITC) assays, we have validated that PDBAG1 is capable of binding C1QBP with a *K*_d_ value of 334 nM. Furthermore, PDBAG1 inhibits homologous recombination repair proteins and facilitates synergism with poly‐ADP‐ribose polymerase inhibitors in cancer therapy. This underscores that PDBAG1 ultimately induces insurmountable survival stress through multiple mechanisms while concurrently engendering therapeutic vulnerabilities specific to TNBC.

**Key points:**

- The newly discovered peptide PDBAG1 is the first small molecule substance found to directly target and degrade C1QBP, demonstrating significant tumour inhibitory effects and therapeutic potential.

## Graphical abstract

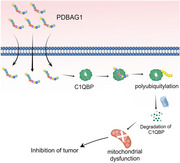

## 1 INTRODUCTION

Triple‐negative breast cancer (TNBC) is characterised by a high propensity for metastasis and poor overall survival.^1^ Given the absence of hormone receptors and human epidermal growth factor receptor 2 expression, TNBC does not respond to endocrine therapy or anti‐HER2 treatment.^2^ Despite limited therapeutic advancements in recent years, chemotherapy remains the cornerstone of TNBC treatment. Considering the scarcity of targeted therapies and unfavourable prognosis, there is an urgent imperative to identify actionable molecular targets and effective strategies for managing patients with TNBC.

C1QBP, also known as p32, is a 32 kDa protein that primarily localises to the mitochondria, cytoplasm and cell membrane. As a trimeric protein, it plays an active role in critical biological processes such as transcriptional regulation, pre‐mRNA splicing, ribosome biogenesis and mitochondrial protein synthesis.^3^ Its importance extends to various disease contexts including inflammation, infection and cancer with a primary focus on mitochondrial regulation.^4^, ^5^ Previous studies have shown increased expression of C1QBP across different malignancies particularly in breast cancer where it serves as a master regulator of mitochondrial homeostasis and metabolism.^6^, ^7^ Aerobic glycolysis, also known as the Warburg effect, has long been considered the predominant energy metabolism in cancer.^8^, ^9^ However, recent evidence challenges this notion by demonstrating that most cancer cells do not solely rely on glycolysis to generate adenosine triphosphate (ATP) due to a permanent impairment of mitochondrial oxidative phosphorylation (OXPHOS; mtOXPHOS). Nevertheless, emerging research suggests that even aggressive tumours can exhibit a metabolic shift towards OXPHOS when genetic disruptions of LDHA/B occur,^10^ which is crucial for tumour growth and progression. Merely inhibiting glycolysis is insufficient for eradicating cancer cells, thereby emphasising the pivotal role of mtOXPHOS in cancer biology. Despite its potential as an anti‐cancer strategy targeting OXPHOS, clinical benefits with OXPHOS inhibitors have yet to be achieved.^11^ Therefore, targeting OXPHOS in various types of cancers including breast cancer represents an attractive therapeutic approach.^12^ The down‐regulation of C1QBP suppresses breast cancer progression but enhances the glycolytic activity of cancer cells,^7^ suggesting that p32 may serve as a promising diagnostic molecule and therapeutic target for maintaining metabolic balance in cancer biology.^13^

As Lyp‐1 (CGNKRTRGC) has been isolated through phage display screening and demonstrated its ability to bind C1QBP at the cell surface,^14^ it has gained significant value in various types of cancer research. It has been successfully prepared in different forms, including radiolabelled, fluorescent and nanoparticle‐based bioconjugates.^15^ Its primary function revolves around the direct regulation of C1QBP activity on the cell surface. The targeting protein degradation (TPD) technology has emerged as a highly promising approach for selectively eliminating disease‐associated proteins by harnessing the cells’ intrinsic machinery for protein destruction.^16^ Currently, no pharmacological agents capable of directly targeting and simultaneously inducing the degradation of C1QBP have been identified.

Peptide drugs can surpass small molecules and larger biologics in certain aspects due to their advantages of target specificity, low toxicity and potential for modification.^17^, ^18^ In this study, we employed peptidomics methods to screen peptides originating from adipose tissue for potential metabolic regulatory functions. Surprisingly, the identification of a novel peptide, PDBAG1, derived from the precursor protein GPD1—an essential metabolic enzyme—has been accomplished. And this newly discovered peptide exhibits significant inhibition of OXPHOS activity. Mechanistically, PDBAG1 directly binds to mitochondrial protein C1QBP and leading to its ubiquitin‐dependent degradation. This process impairs OXPHOS and DNA repair mechanisms, while simultaneously reducing the capacity for glycolytic reserve. Through size‐exclusion chromatography (SEC) and isothermal titration calorimetry (ITC) assays, we validated that PDBAG1 is capable of binding C1QBP with a *K*_d_ value of 334 nM. Consequently, it induces insurmountable survival stress through multiple pathways and creates therapeutic vulnerabilities for TNBC. Upon treatment with PDBAG1, MDA‐MB‐231 and MDA‐MB‐468 cells exhibited increased glycolysis as an adaptive response to maintain ATP levels. Notably, similar elevation in glycolysis has been observed upon inhibition of mitochondrial complex I by metformin.^19^ However, PDBAG1 did not alter the overall glycolytic capacity but rather further decreased the glycolytic reserve capacity—thus limiting metabolic flexibility to some extent. Additionally, PDBAG1 inhibited the DNA damage repair pathway involving BRCA1/2 and RAD51/54 proteins. Suppression of BRCA1 expression induced a ‘BRCAness’ phenotype that synergised with poly‐ADP‐ribose polymerase (PARP) inhibitors. Therefore, PDBAG1 may serve as a new inhibitor of C1QBP by targeting mtOXPHOS activity, while also modulating the capability of homologous recombination (HR) repair.

## 2 RESULTS

### 2.1 New peptide PDBAG1 and its in vitro and in vivo anti‐cancer effect in breast cancer

**FIGURE 1:**
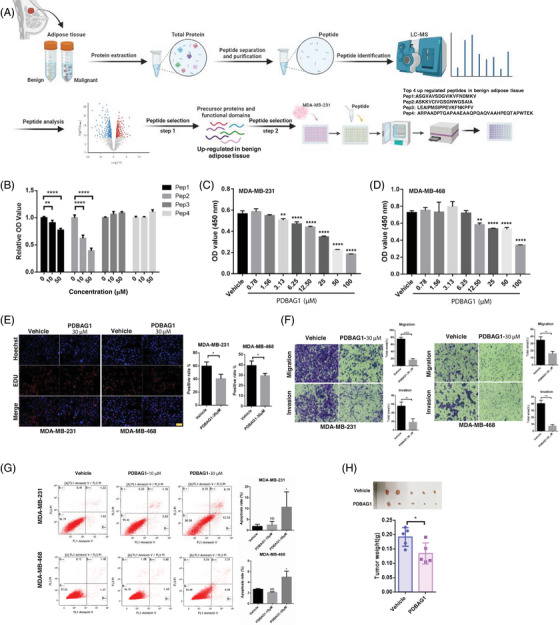
New peptide PDBAG1 exhibits inhibition function in TNBC in vitro and in vivo. (A) Schematic diagram of the screening process of endogenous anti‐tumour peptide PDBAG1. (B) Relative OD values were detected after 48 h of treatment with different concentrations of four peptides in MDA‐MB‐231cells (*n* = 4). **p < .01, ****p < .0001, compared with vehicle group. (C and D) OD values were detected after 48 h of treatment with different concentrations of PDBAG1 in MDA‐MB‐231and MDA‐MB‐468 cells (*n* = 4). *p < .05, **p < .01, ****p < .0001, compared with vehicle group. (E) The cell EdU positive rate was detected in MDA‐MB‐231 and MDA‐MB‐468 cells after cultured with different concentrations of PDBAG1 for 48 h. Scale bar, 50 µm. (F) Transwell assay was performed on MDA‐MB‐231 and MDA‐MB‐468 cells lines after PDBAG1(30 µM) treatments. (G) Cell death was performed and quantified by fluorescence‐activated cell sorter (FACS) analysis on MDA‐MB‐231 and MDA‐MB‐468 cells that stained annexin V/PI after 24 h PDBAG1(30 µM) treatments. *p < .05, compared with vehicle group. (H) The photo of xenografted mice model treated with PDBAG1 every 2 days and the tumour weights were measured in each group after sacrifice of xenograft mice at the 21th day. *n* = 5. *p < .05, **p < .01, ****p < .0001, compared with vehicle group; Student's *t*‐test. Error bars, mean ± SD.

Peptide drugs, although currently a small category of pharmaceutical molecules, are expanding rapidly. Researchers have employed diverse approaches to discover and modify peptides with potential therapeutic value across various domains. In this study, we utilised liquid chromatography–mass spectrometry (LC–MS) to identify potential endogenous anti‐cancer peptides from different tissues. Cell‐penetrating peptides (CPPs), recognised as effective facilitators of intracellular delivery, possess the capability to transport various conjugates, including peptides, into the cytosolic space of cells, thereby exhibiting valuable biologically active properties.^20^ The process of functional peptide screening is depicted (Figure 1A). Top four peptides highly expressed in benign breast adipose tissue and located within the functional domain of precursor proteins were selected for synthesis. After treatment of tumour cells with these peptides, CCK‐8 assay was used to detect the effects of these peptides on tumour proliferation. Fortunately, we identified a new peptide (ASKKVCIVGSGNWGSAIA) derived from glycerol‐3‐phosphate dehydrogenase 1 (GPD1) protein, which was conjugated with CPP (GRKKRRQRRRPPQQ). Remarkably, this modified peptide significantly suppressed the cell viability of MDA‐MB‐231 (Figure 1B). Furthermore, the inhibitory effects on both MDA‐MB‐231 and MDA‐MB‐468 cells were substantial and exhibited dose dependency (Figure 1C,D). We designated this engineered peptide as PDBAG1 (peptide derived from breast adipose tissue GPD1: GRKKRRQRRRPPQQASKKVCIVGSGNWGSAIA; Figure S1A), which precursor (ASKKVCIVGSGNWGSAIA) displayed high expression levels in benign adipose tissue and was found within the functional domains of its precursor protein GPD1. As illustrated (Figure S1B), PDBAG1 demonstrated potent cell‐penetrating properties in both MDA‐MB‐231 and MDA‐MB‐468 cells. Subsequently, we conducted an extensive investigation into the functionality of PDBAG1 in TNBC cancer cells and discovered that it significantly impeded proliferation, invasion and migration while modestly promoting apoptosis in both MDA‐MB‐231 and MDA‐MB‐468 cell lines as depicted (Figure 1E–G). Additionally, our cell experiments have demonstrated that PDBAG1 exhibits significant inhibitory effects on various cell lines including murine TNBC cell lines (E0771 and 4T1; Figure S1C,D). These significant inhibitory effects were observed at concentrations around 30 µM (Figure S1E). To evaluate the anti‐cancer properties of PDBAG1 when administered in vivo for TNBC treatment purposes, we established tumour xenografts using human TNBC cell line MDA‐MB‐231 implanted into nude mice. The results presented (Figure 1H) clearly demonstrate that treatment with PDBAG1 resulted in a significant reduction in tumour weight compared with control groups. These findings strongly suggest that PDBAG1 may effectively inhibit cancer cell growth in vitro and induce tumour regression in vivo in a TNBC xenograft model.

### 2.2 PDBAG1 induces mitochondria dysfunction and OXPHOS inhibition

Considering that: (1) the peptide PDBAG1 was identified from adipose tissue, a crucial regulator of energy homeostasis; (2) it is derived from GPD1, an essential enzyme in cellular metabolism; (3) it is located within the functional domains of the precursor protein GPD1. These collective findings suggest a potential association between the inhibitory effects of peptide PDBAG1 on breast cancer and cancer metabolism. Unlike normal cells, tumour cells generate their total intracellular ATP through two energetic pathways, namely glycolysis and mtOXPHOS.

**FIGURE 2:**
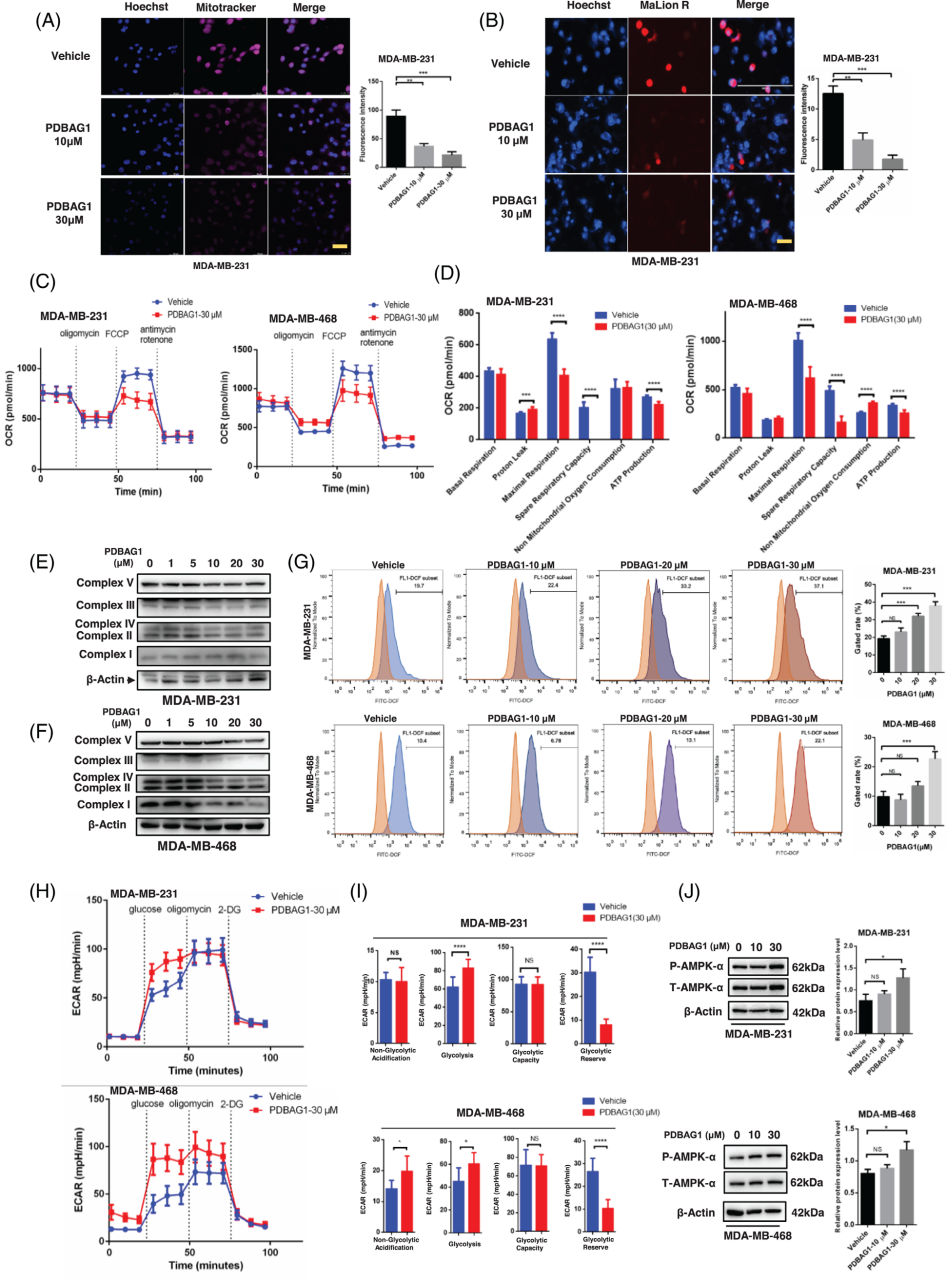
PDBAG1 induces mitochondria dysfunction and oxidative phosphorylation inhibition in TNBC cells. (A) Changes in mitochondrial mass (mitotracker) after PDBAG1 treatment were assessed by confocal microscopy (Leica STELLARIS STED). Quantification from three independent experiments is presented as histograms. Magnification: 40×, scale bar: 50 µm (**p < .01, ***p < .001). (B) The cellular ATP levels were measured in MDA‐MB‐231 cells following treatment with vehicle and PDBAG1, using the MalionR ATP sensor. Quantification from three independent experiments is presented as histograms. Magnification: 40×, scale bar, 50 µm (EVOS FL AUTO, Life, **p < .01, ***p < .001). (C and D) Seahorse XF24 was used to detect mitochondrial stress after PDBAG1 treatment on MDA‐MB‐231 and MDA‐MB‐468 cells, the basal respiration, proton Leak, maximal respiration, spare respiratory capacity, non‐mitochondrial oxygen consumption, ATP production in each group were measured (***p < .001, ****p < .0001, compared with vehicle group; Student's *t*‐test. Error bars, mean ± SD, MDA‐MB‐231 *n* = 12 and MDA‐MB‐468 *n* = 9). (E and F) Protein levels of mitochondrial complexes after treatment with different concentrations of PDBAG1 in TNBC cell lines were detected by immunoblotting. (G) Intracellular ROS levels were detected by flow cytometry after PDBAG1 treatment of TNBC cells for 24 h. A bar graph shows the percentage of gated cells, quantifying ROS levels across treatment groups (***p < .001, compared with vehicle group; Student's *t*‐test. Error bars, mean ± SD, *n* = 3). (H and I) Seahorse XF24 was used to detect glycolytic stress after PDBAG1 treatment on TNBC cell lines, the non‐glycolytic acidification, glycolysis, glycolytic capacity, glycolytic reserve in each group were measured (NS, non‐significant, *p < .01, ****p < .0001, compared with vehicle group; two‐way ANOVA test. Error bars, mean ± SD, *n* = 12). (J) Western blot analysis was employed to assess alterations in AMPK phosphorylation levels within TNBC cells following a 24‐h treatment with PDBAG1.

First, we assessed the impact of peptides on mitochondria. The fluorescence intensity of mitotracker, a commonly used dye for measuring mitochondrial content, was significantly reduced in the PDBAG1‐treated group, indicating altered mitochondrial structure (Figures 2A and S2A). ATP serves as a pivotal regulator of cellular metabolism. We assessed the impact of peptides on intracellular ATP production. To investigate the energy metabolism in PDBAG1‐treated MDA‐MB‐231 cells, the MaLionR plasmid was utilised to directly interact with the intracellular ATP content. Following transfection, a significant reduction in fluorescence intensity was observed after treating the cells with the peptide for 24 h. As depicted (Figures 2B and S2B,C), the PDBAG1‐treated group exhibited a more pronounced reduction in intracellular ATP content compared with the control group which showed only marginal changes. These findings suggest that PDBAG1 impairs OXPHOS‐dependent metabolism. Similar inhibitory effects were observed in MDA‐MB‐468 cells where PDBAG1 significantly suppressed both glycolytic and oxidative energy metabolism (Figure 2C). Collectively, these results demonstrate that PDBAG1 exerts substantial inhibition on mtOXPHOS‐mediated ATP generation. Importantly, oxygen consumption rate (OCAR) analysis further revealed that OXPHOS was significantly inhibited in MDA‐MB‐231 and MDA‐MB‐468 cells treated with PDBAG1 (Figure 2C). Maximal respiration, spare respiratory capacity and ATP production were all significantly inhibited by PDBAG1 in both cell lines (Figure 2D). Furthermore, OXPHOS complexes were impaired to varying degrees by PDBAG1 treatment (Figure 2E,F), including inhibition of ATP synthase subunit alpha of Complex V (ATP5A) which directly generates ATP. Reactive oxygen species (ROS) play a crucial role in various biological processes and mitochondria are the main source of ROS within cells. Mitochondrial dysfunction is often associated with increased ROS production from these organelles.^21^ Consistently, we observed an increase in ROS levels in both MDA‐MB‐231 and MDA‐MB‐468 cells treated with PDBAG1, indicating mitochondrial dysfunction caused by PDBAG1 leading to impaired energy metabolism (Figure 2G). Consequently, this results in decreased ATP production and increased ROS generation, which may contribute to the inhibition of TNBC growth and tumour regression. Cancer cells often adapt their metabolism to survive under environmental stress conditions by switching between glycolysis and OXPHOS pathways.^22^ Therefore, we further investigated the impact of PDBAG1 on glycolytic stress by monitoring extracellular acidification rate in TNBC cell lines. As depicted (Figure 2H,I), treatment with PDBAG1 induced an elevation in glycolytic rate but reduced the glycolytic reserve capacity in both tested MDA‐MB‐231 and MDA‐MB‐468 cells. However, it did not affect the glycolytic capacity while significantly decreasing the glycolytic reserve capacity to some extent, suggesting limited metabolic flexibility. The phosphorylation levels of AMPK in TNBC cells were evaluated using Western blot analysis after a 24‐h treatment with PDBAG1 at 30 µM. The observed alterations suggest disruptions in cellular energy metabolism, indicating the induction of a possible metabolic stress^23^ (Figure 2J).The CCK‐8 assay quantifies the difference in absorbance of substances metabolised by mitochondrial dehydrogenases at 450 nm. Our study in this paper discovered that PDBAG1 can induce mitochondrial dysfunction, and there were slight variations in mitochondrial function between the two cell lines. Under the same concentration of PDBAG1, MDA‐MB‐468 cells produced more ROS (Figure 2G), suggesting potential alterations in mitochondrial dehydrogenases status that could impact the CCK‐8 experiment. Therefore, the results of the CCK‐8 assay only partially reflect PDBAG1's inhibitory effect on tumour cell growth. Additionally, we observed significant deterioration in morphology and state of MDA‐MB‐468 cells after 24‐h treatment with PDBAG1 (Figure S2D). In the clone formation experiment, 30 µM PDBAG1 rendered ineffective clone formation for MDA‐MB‐468 cells (Figure S2E).

### 2.3 PDBAG1 induces hypoxia response under normal oxygen conditions

**FIGURE 3:**
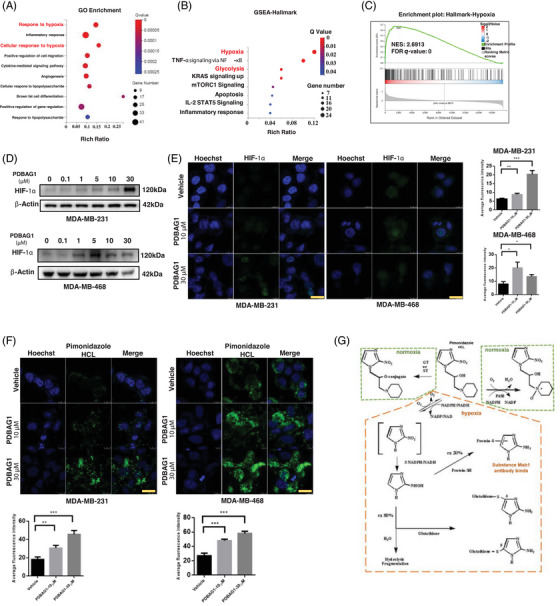
PDBAG1 induces hypoxia, promotes glycolysis in TNBC cells. (A and B) GO enrichment analysis and GSEA analysis were performed by GOseq on differential expressed genes (foldchange ≥1.5) between vehicle and PDBAG1 treated samples (*n* = 3). (C) GSEA enrichment analysis in Hallmark dataset of hypoxia (NES = 2.6913, FDR *q* value = 0). (D) HIF‐1α level was detected by immunoblotting after treated with different concentrations of PDBAG1. (E) Immunofluorescence was used to detect the expression of HIF‐1α in TNBC cells treated with PDBAG1. Quantification from three independent experiments is presented as histograms. Magnification: 40×, scale bar, 20 µm (Leica STELLARIS STED). (F) Extent of intracellular hypoxia was detected by using hypoxy probe after PDBAG1 treatment. Quantification from three independent experiments is presented as histograms. Magnification: 40×, scale bar, 20 µm (Leica STELLARIS STED). (G) Schematic illustration depicting the operational principle of hypoxy probe (pimonidazole HCL), which can undergo a reaction with sulphide under hypoxic conditions to generate an adduct capable of binding monoclonal antibodies.

To elucidate the potential molecular mechanism underlying the anti‐cancer effects of PDBAG1 against TNBC, we employed RNA sequencing (RNA‐seq) to profile MDA‐MB‐231 cells after 24 h of PDBAG1 treatment (Figure S3A–C). Notably, hallmark‐annotated gene set‐enrichment analysis (GSEA) revealed a significant inhibition of OXPHOS in PDBAG1‐treated cells (Figure S3D). This finding is consistent with previous results demonstrating that PDBAG1 induces mitochondrial dysfunction and inhibits OXPHOS. Furthermore, GO, KEGG pathway enrichment and GSEA robustly confirmed that peptide PDBAG1 induced a hypoxia response (Figure 3A–C). It is well established that hypoxia stabilises and accumulates HIF‐1α protein. Consistent with this notion, as illustrated that treatment with PDBAG1 markedly increased the protein level of HIF‐1α in MDA‐MB‐231 cells, especially at a concentration of 30 µM (Figure 3D). Notably, the up‐regulation of HIF‐1α in MDA‐MB‐468 cells was observed to be mild at a concentration of 30 µM. This observation aligns with the previous findings depicted (Figures 2G and S2A), where MDA‐MB‐468 cells exhibited more mitochondria inhibition and ROS production. The immunofluorescence results presented (Figure 3E) were consistent with those obtained from western blot analysis, both indicating an increase in intracellular HIF‐1α levels following treatment with PDBAG1 (30 µM in MDA‐MB‐231 and 10 µM in MDA‐MB‐468). Furthermore, GSEA analysis revealed that glycolysis emerged as one of the most prominent hallmarks when TNBC was subjected to PDBAG1 treatment (Figure 3B). Our previous experiments utilising seahorse assays have also confirmed that peptide treatment promotes glycolysis (Figure 2H). In hypoxic conditions, it is well established that many cancer cells shift their primary metabolic strategy from predominantly relying on mitochondrial respiration towards increased reliance on glycolysis for ATP production.^24^ To further validate the induction of hypoxia by PDBAG1, we assessed intracellular hypoxia by measuring the intensity of a hypoxy probe following treatment. Our results demonstrated significantly higher fluorescence levels of pimonidazole HCL in both MDA‐MB‐231 and MDA‐MB‐468 cells treated with PDBAG1 compared with controls, particularly at a concentration of 30 µM (Figure 3F). The principle of the hypoxia probe is based on the competition between O_2_ and pimonidazole for the first electron. In hypoxic cells (oxygen concentration below 1.3% or oxygen partial pressure threshold ≤10 mm Hg), pimonidazole can be reduced and activated in the presence of hypoxia. The activated intermediate then forms stable adducts by reacting with thiol groups in proteins, peptides and amino acids (Figure 3G). These adducts are detected using immunochemical methods through binding with Mab1 (anti‐pimonidazole HCL) antibody.^25^ Considering that these cell experiments were all conducted under normal oxygen conditions, it is unlikely for the oxygen partial pressure within cells to significantly decrease. It can be inferred from the action principle of pimonidazole that there may exist high concentrations of reducing substances in cells which bind to pimonidazole, leading to ‘pseudo‐hypoxia’ under normal oxygen conditions. Treatment with PDBAG1 resulted in a decrease in hydrox‐HIF‐1α, as expected. Additionally, it down‐regulated the expression of FIH (factor inhibiting HIF‐1) and PHD2 proteins. These findings suggest that PDBAG1 up‐regulates the HIF‐1α and HIF‐2α subunit by inhibiting its hydroxylation (Figure S3E). The increase in ROS levels after PDBAG1 treatment could leads to the down‐regulation of FIH and PHD2, which in turn results in decreased hydroxylation of HIFs, thereby stabilising them. The HIF‐1 transcription factor plays a crucial role in survival stress regulation and activates various genes associated with tumour cell survival, particularly glycolysis. However, there was only a slight increase or even a decrease observed in the expression of HIF‐1α target genes after PDBAG1 treatment in MDA‐MB‐231 cells (Figure S3F,G). This may indicate that up‐regulation of HIF‐1α fails to effectively activate its transcriptional function. It is important to note that while HIF‐1β is constitutively expressed and stable, the expression of HIF‐1α can be flexibly regulated. Interestingly, our study showed that PDBAG1 reduced the expression of HIF‐1β (Figure S3H) in MDA‐MB‐231 cells.

### 2.4 PDBAG1 directly binds C1QBP and further reduces the protein

**FIGURE 4:**
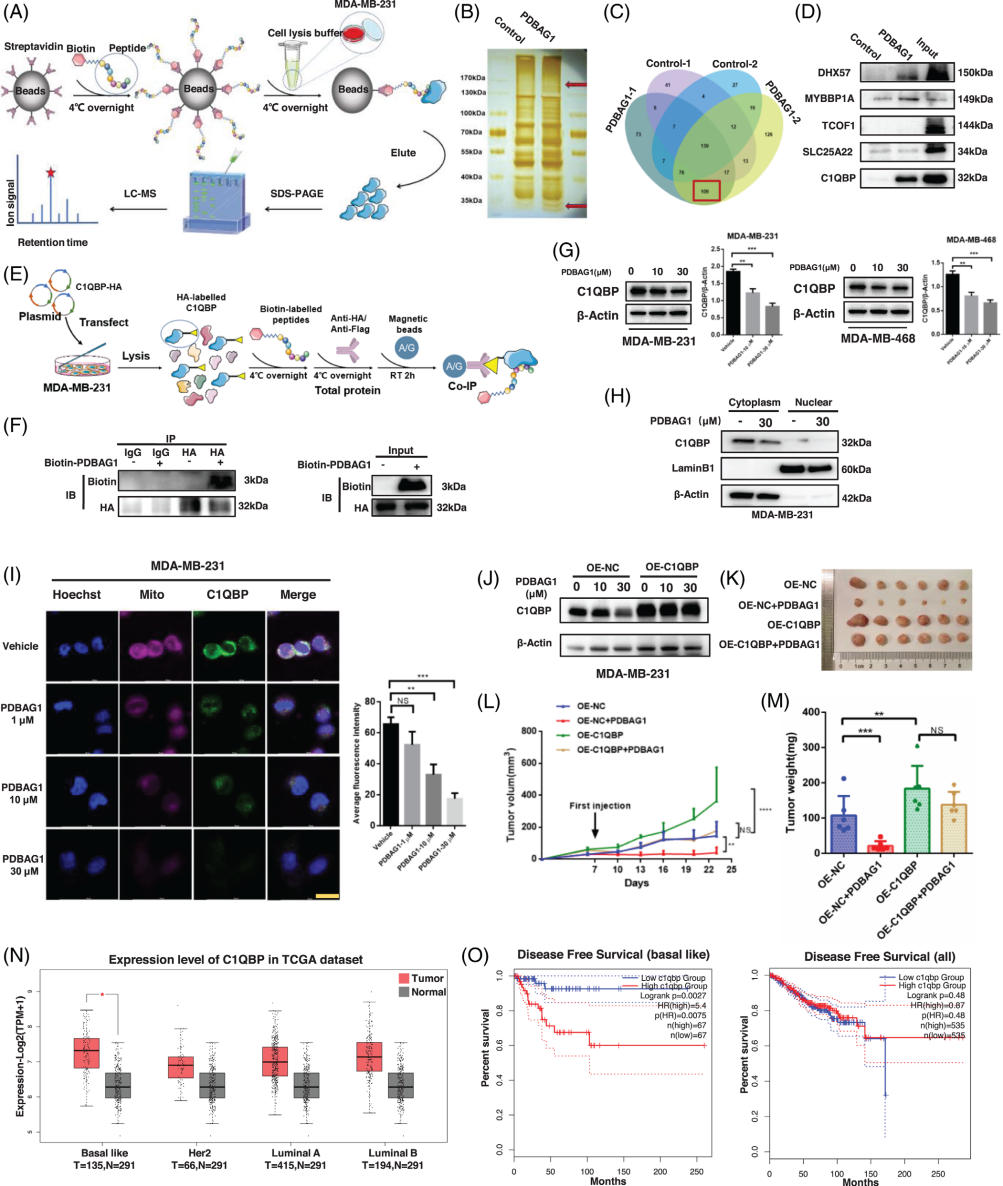
PDBAG1 inhibits TNBC by direct target and decrease C1QBP at protein level. (A) Schematic diagram of the biotin‐labelled PDBAG1 pull down assay. (B and C) The silver staining gel picture and mass spectrometry results of the pulldown assay. (D) Pulldown experiment to verify the top five high abundant proteins bound by PDBAG1. (E and F) Schematic diagram and the results of the co‐IP assay (HA‐tagged C1QBP were used as bait, and biotin‐labelled PDBAG1 was added to the protein lysate). (G and H) Protein level of C1QBP was tested in TNBC cell lines after treated with PDBAG1 and change of C1QBP in the cytoplasm and nucleus of MDA‐MB‐231 cells after treated with PDBAG1. (I) 24 h after MDA‐MB‐231 cells were treated with PDBAG1, the intracellular fluorescence intensity and distribution of C1QBP and mitotracker (Thermo Fisher) were detected by immunofluorescence. Quantification of C1QBP from three independent experiments is presented as histograms. Magnification: 40×, Scale bar, 20 µm (Leica STELLARIS STED). (J) Stable overexpression C1QBP MDA‐MB‐231 cell lines were constructed to detect C1QBP protein level after treated with PDBAG1. (K, L and M) Stable overexpression tumour cells were inoculated into nude mice for tumourigenic model experiments (*n* = 6 mice in each group) were constructed, after treated with vehicle or PDBAG1 the effects were shown on tumour image, tumour volume and tumour weight were examined (NS, non‐significant, *p < .05, **p < .01, ***p < .001; two‐way ANOVA test. Error bars, mean ± SD, *n* = 6). (N and O) The expression levels of C1QBP in different subtypes of breast cancer in the TCGA database and the effect of high expression of C1QBP on DFS of patients with all types of breast cancer and triple negative breast cancer (GEPIA2, http://gepia2.cancer‐pku.cn).

To elucidate the precise mechanism underlying the inhibitory effect of PDBAG1 on OXPHOS, we employed a peptide–protein pull‐down assay coupled with LC–MS to identify proteins interacting with PDBAG1 (Figure 4A). Through careful selection and exclusion, we identified 109 PDBAG1‐binding proteins in silver‐stained gel strips (Figure 4B,C). By analysing the potential binding proteins, we have identified their involvement in crucial biological processes such as post‐translational modification, intercellular signalling, and cellular metabolism (Figure S4A,B). Based on mass spectrometry results, expression abundance and molecular weight positioning of cut gel strips (around 150 and 30 kDa), DHX57, MYBBP1, TCOF1, SLC25A22 and C1QBP were chosen for validation of their binding to PDBAG1. Pull‐down assays confirmed that both DHX57 and C1QBP interacted with PDBAG1 (Figure 4D), particularly C1QBP. Among these enriched proteins, our attention was drawn to C1QBP due to its established role in mitochondrial metabolism. To gain further insights into the interactions between C1QBP and PDBAG1, anti‐biotin immunoprecipitation assays were conducted. Following the experimental procedure depicted (Figure 4E), we observed that C1QBP acted as bait and bound to PDBAG1 (Figure 4F). Given the binding ability of PDBAG1 with C1QBP demonstrated earlier, we subsequently analysed the protein expression levels of C1QBP in TNBC cell lines treated with different concentrations of PDBAG1 (10 and 30 µM). Our findings revealed a significant inhibition of C1QBP expression at the protein level upon treatment with both concentrations of PDBAG1 compared with control groups (Figures 4G and S4C,D). Furthermore, our analyses of TNBC cells treated with PDBAG1 indicated no significant impact on the protein levels of DHX57(Figure S4I). The subcellular localisation is crucial for proper functioning of both forms.^3^, ^26^ Therefore, the nuclear‐cytoplasmic fractionation assay revealed that C1QBP was widely expressed with a predominant cytoplasmic localisation, which was also observed in nuclear immunoblotting. Specifically, we observed a significant reduction of cytoplasmic C1QBP upon treatment with PDBAG1 (Figure 4H). To investigate the direct binding of PDBAG1 to C1QBP in both the cytoplasm and nucleus, we further extracted proteins from these compartments for peptide pull‐down assays followed by immunoblotting analysis. As depicted (Figure S4E), our results indicated that biotin‐PDBAG1 could interact with C1QBP in both the cytoplasm and nucleus. Moreover, immunofluorescent staining was employed to validate that PDBAG1 induced changes in mitochondrial mass through down‐regulation of C1QBP protein levels (Figure 4I). In addition, PDBAG1 also down‐regulates C1QBP expression levels in both murine TNBC cell lines (4T1 and E0771; Figure S4F) and blood tumour cell lines (Jurkat and THP‐1; Figure S4G,H).

### 2.5 C1QBP mediates PDBAG1‐induced TNBC tumour regression

To further validate the inhibitory effect of PDBAG1 on TNBC through C1QBP protein reduction, we generated an MDA‐MB‐231 cell line overexpressing C1QBP (Figure 4J) to rescue the impact of PDBAG1 (Figure S4J). In order to investigate the in vivo effects of PDBAG1 and C1QBP, we employed an orthotopic breast cancer tumourigenesis nude mice model for validation. MDA‐MB‐231 cells stably overexpressing vector and C1QBP were transplanted into nude mice mammary fat pads, and tumour volume as well as body weight of nude mice were measured every 3 days starting from the 6th day post‐transplantation. From the 7th day onwards, intraperitoneal injections of PDBAG1 at a dosage of 10 mg/kg were administered every 3 days. The results demonstrated that PDBAG1 significantly inhibited TNBC growth in vivo, with this effect being reversed by C1QBP (Figure 4K–M). Furthermore, the inhibitory effect of PDBAG1 on cells was significantly attenuated following stable knockdown of C1QBP induced by doxycycline, as demonstrated by the CCK8 assay (Figure S4K). Additionally, we analysed the expression and prognostic value of C1QBP using TCGA breast cancer data. We observed up‐regulated mRNA expression levels of C1QBP exclusively in TNBC tumour tissues compared with normal adjacent tissue (Figure 4N), while no significant differences were found in other subtypes of breast cancer tumours including Her2‐overexpression, luminal A and luminal B types. High mRNA expression levels of C1QBP were significantly associated with poor disease‐free survival (DFS) specifically in patients with TNBC; however, its prognostic value was not significant across all breast cancer patients (Figure 4O). These findings indicate that C1QBP is highly expressed in TNBC and can be targeted by our peptide PDBAG1, thereby providing a potential therapeutic target and new treatment strategies for TNBC.

### 2.6 PDBAG1 binds C1QBP and promotes ubiquitin‐dependent degradation, which further mediates mitochondrial dysfunction

**FIGURE 5:**
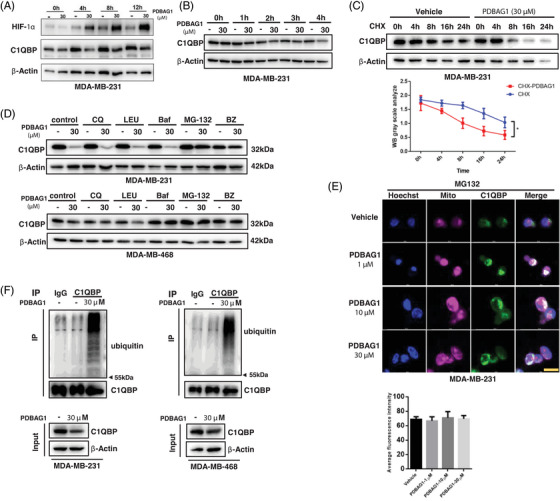
PDBAG1 promotes ubiquitin‐dependent degradation of C1QBP. (A) The protein level of C1QBP and HIF‐1α were assessed by immunoblotting in TNBC cells after treated with PDBAG1 within 12 h. (B) C1QBP protein level was assessed by immunoblotting after treated with PDBAG1 within 4 h. (C) The degradation speed of C1QBP protein was performed with protein extracted at 0, 4, 8, 16 and 24 h after treatment of MDA‐MB‐231 cells with cycloheximide (CHX) and PDBAG1. (D) Treating TNBC cells with inhibitors of different protein degradation associated pathways stacked with PDBAG1, the ubiquitin–proteasome pathway inhibitors MG‐132 and BZ could reverse the decrease in C1QBP protein level (long expose) caused by PDBAG1 (CQ, chloroquine; LEU, leupeptin hemisulphate; Baf, bafilomycin A1; BZ, bortezomib). (E) 24 h after MDA‐MB‐231 cells were treated with PDBAG1 and MG132, the intracellular fluorescence intensity and distribution of C1QBP and mitotracker (thermos fisher) were detected by immunofluorescence. Quantification from three independent experiments is presented as histograms. Magnification: 40×, Scale bar, 20 µm (Leica STELLARIS STED). (F) The impact of PDBAG1 treatment on C1QBP ubiquitination was assessed over a 12‐h period using co‐immunoprecipitation (co‐IP) assays. Results demonstrate a significant enhancement of C1QBP ubiquitination upon exposure to PDBAG1.

PDBAG1 significantly suppresses the expression of C1QBP, while its transcript remains unaffected. The decrease in C1QBP is concentration‐dependent and rapid, with remarkable effects observed within 4 h (Figure 5A,B). And the results demonstrated a simultaneous decrease in C1QBP and an increase in HIF‐1α upon exposure to PDBAG1. To investigate whether PDBAG1 affects C1QBP expression, we added cycloheximide to inhibit protein synthesis. As expected, the degradation of C1QBP protein was accelerated in MDA‐MB‐231 cells treated with PDBAG1 compared with the control group (Figure 5C). These findings suggest that PDBAG1 promotes the degradation of C1QBP.

To elucidate the mechanisms underlying the decrease in C1QBP levels, we treated MDA‐MB‐231 and MDA‐MB‐468 cells with various inhibitors: autophagy inhibitor chloroquine (CQ), leupeptin hemisulphate (LEU), bafilomycin A1 (Baf), proteasome inhibitor MG‐132 and bortezomib (BZ) along with PDBAG1 treatment. The reduction in C1QBP expression could be largely rescued by both MG‐132 and BZ, two specific proteasome inhibitors, but not by CQ, LEU or Baf treatment alone (Figure 5D). Furthermore, immunofluorescent staining confirmed that MG‐132 could inhibit the degradation of C1QBP induced by PDBAG1 and restore mitochondrial function (Figure 5E). Therefore, these results indicate that PDBAG1 promotes ubiquitin‐dependent degradation of C1QBP through the ubiquitin–proteasome system rather than autophagic degradation. The impact of PDBAG1 treatment on C1QBP ubiquitination was assessed over a 12‐h period using co‐immunoprecipitation (co‐IP) assays (Figure 5F). The ubiquitination modification of C1QBP was detected in both MDA‐MB‐231 and MDA‐MB‐468 cells. Since the 26S proteasome has a binding preference for ubiquitin chains containing four or more Ubiquitin molecules, the ubiquitination‐modified band corresponding to modified‐C1QBP appears above 55 kDa on SDS‐PAGE gels.

### 2.7 Validation of binding of PDBAG1 and C1QBP

**FIGURE 6:**
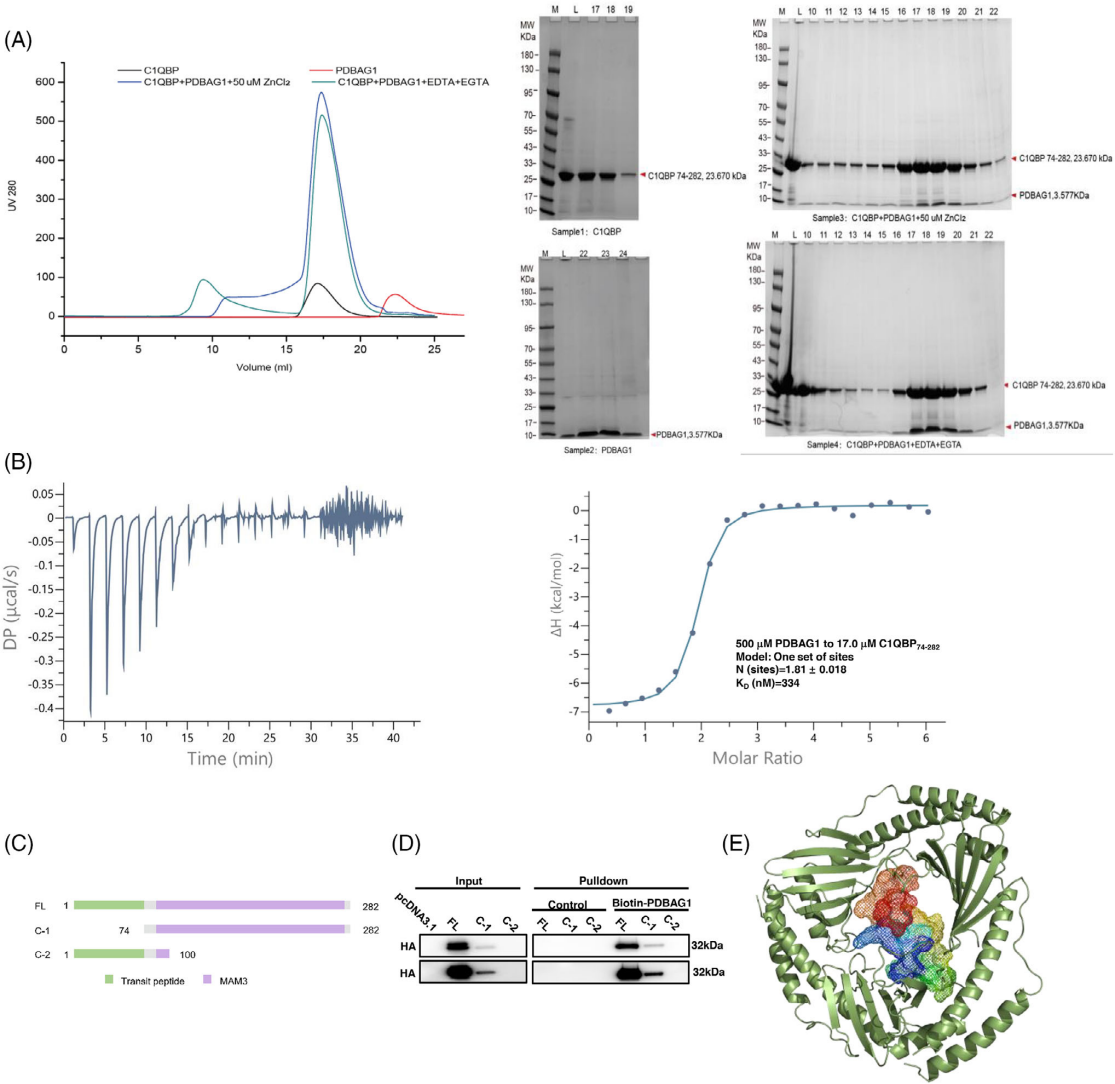
PDBAG1 directly binds to C1QBP protein. (A) Gel filtration elution profiles for PDBAG1 (red), C1QBP (black), PDBAG1+C1QBP (green) and PDBAG1+C1QBP+ZnCL_2_ (blue) are shown on the left(C1QBP to PDBAG1 ratio = 1:1.2), and Coomassie‐stained SDS‐PAGE gels of the fractions collected are shown on the right. Co‐elution of C1QBP and PDBAG1 complexes was detected, indicating the presence of binding of C1QBP and PDBAG1, while addition of EDTA and EGTA without Zn^2+^ had the same phenomenon, suggesting that binding may not be metal ion‐dependent. (B) Isothermal titration calorimetry (ITC) of peptide (500 µM) into C1QBP_74–282_ (17.0 µM) in 20 mM potassium phosphate, 100 mM NaCl, pH 7.4. It was showed that the experimentally derived curve with raw heat rate/μcal s^−1^ versus time/min (*left*) and the calculated binding isotherm with change in enthalpy/kcal mol^−1^ versus C1QBP_74–282_–PDBAG1 molar ratio (*right*, *K*_d _= 334 nM). (C and D) Schematic diagram of the construction of the truncated form of C1QBP and the results of pulldown experiments between PDBAG1 and C1QBP truncated forms. (E) Molecular docking model of C1QBP (PDB: 1P32) and PDBAG1.

Gel filtration was employed to investigate the binding state of C1QBP and PDBAG1, with a previous report indicating that the presence of Zn^2+^ might be necessary for C1QBP binding. When we applied in a 1:1.2 ratio of C1QBP to PDBAG1 and mixed with 50 µM Zn^2+^, co‐elution of the C1QBP and PDBAG1 complex was observed (Figure 6A). Simultaneous addition of EDTA and EGTA without Zn^2+^ resulted in similar complex efflux, suggesting that the binding may not rely on metal ions. To further explore this, we utilised ITC analysis and demonstrated that C1QBP can bind to PDBAG1 even in the absence of Zn^2+^ (*K*_d_ = 334 nM; Figure 6B). In order to elucidate the specific domains involved in PDBAG1 binding, two truncations of C1QBP were constructed: 1–100AA and 74–282AA as depicted (Figure 6C). Each fragment was fused with HA at its C‐terminus for pull‐down analyses. Upon transfection into MDA‐MB‐231 cells, cellular protein lysates containing each truncate were incubated with PDBAG1. The structure of C1QBP is relatively straightforward comprising a mitochondrial localisation sequence at its N‐terminal domain. Our results indicate that the functional region responsible for binding lies within residues from position 74 to position 282AA within the N‐terminal domain of C1QBP protein (Figure 6D). Similarly using an online prediction tool H‐dock (http://hdock.phys.hust.edu.cn/), we found consistent results showing that PDBAG1 can bind specifically to these same domains on C1QBP despite forming a homotrimer (Figure 6E).

### 2.8 PDBAG1 suppresses HR repair by repressing BRCA1/RAD54 expression in TNBC cells

**FIGURE 7:**
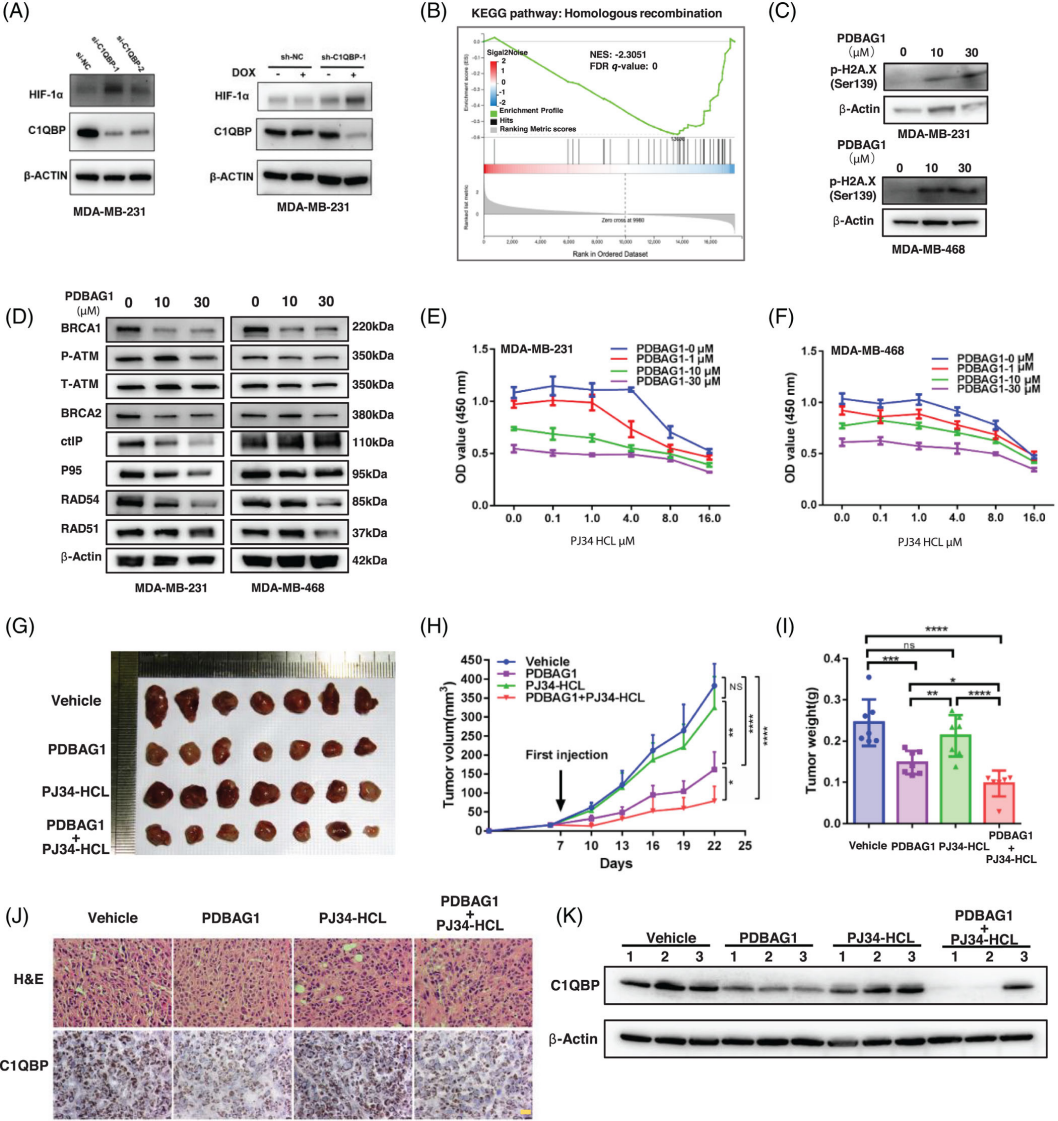
PDBAG1 suppresses homologous recombination repair and induces PARP inhibitor sensitivity in vitro and in vivo. (A) The changes in HIF‐1α protein were observed by Western blot analysis following both transient and long‐term C1QBP knockdown. (B) GSEA enrichment analysis results in KEGG‐pathway dataset of homologous recombination (NES = −2.3051, FDR *q* value = 0). (C) The protein level of phospho‐H2A.X(Ser139) after treated with PDBAG1 in TNBC cell lines. (D) Expression of homologous recombination pathway related proteins after PDBAG1 treatment for 24 h on TNBC cell lines. (E and F) TNBC cells were treated with corresponding concentrations of PDBAG1 and PJ34‐HCL for 24 h in MDA‐MB‐231 and MDA‐MB‐468 cells, and the OD value of the treated cells was measured by CCK8 method. (G, H and I) Using vehicle, PDBAG1, PJ34‐HCL and PDBAG1 combined with PJ34‐HCL administration in nude mice tumourigenic model experiments (*n* = 7 mice in each group), the effects of these administrations on tumour image, tumour volume and tumour weight were examined (NS, non‐significant, *p < .01, **p < .001, ***p < .0001, ****p < .00001, two‐way ANOVA test. Error bars, mean ± SD, *n* = 7). (J and K) The expression of C1QBP in nude mice tumourigenic model after administration was detected by immunohistochemistry and western blot, Magnification: 20×, Scale bar, 20 µm.

The effect of C1QBP knockdown on cellular response was investigated, revealing a significant increase in HIF‐1α protein levels upon C1QBP depletion. These findings are consistent with the observed elevation of HIF‐1α induced by PDBAG1 (Figure 7A). Recent study suggest C1QBP could promote HR repair by forming a complex with MRE11 and RAD50.^27^ Consistent with this finding, it is noteworthy that the HR repair pathway is significantly enriched according to KEGG analysis (Figure 7B; NES: ‐2.3051, FDR *q* value: 0). Notably, hypoxia has been closely associated with DNA repair processes, particularly HR repair under hypoxic conditions.^28^ Phospho‐H2AX or gamma‐H2AX serves as a marker for DNA double‐stranded breaks and can be utilised to assess DNA damage levels.^29^ As anticipated, PDBAG1 treatment induced substantial DNA damage in our experiments (Figure 7C). To further investigate whether PDBAG1‐induced hypoxia leads to alterations in the HR repair pathway and potential inhibition of DNA repair pathways by PDBAG1 treatment group. Consequently, we hypothesise that PDBAG1 treatment significantly inhibits DNA repair mechanisms thereby posing a threat to TNBC cell survival. Our results demonstrate significant down‐regulation of major HR‐related proteins such as BRCA1/BRCA2, RAD54 and RAD51 in MDA‐MB‐231 and MDA‐MB‐468 cells treated with PDBAG1, especially BRCA1 and RAD54. The presence of BRCA mutations is relevant in the context of therapeutic strategies based on PARP inhibitors (PARPis); notably, there was a decrease observed in BRCA protein levels subsequent to PDBAG1 treatment (Figure 7D). Therefore, PDBAG1 could suppress BRCA‐mediated HR repair deficiency leading to sensitisation of TNBC cancer cells without BRCA mutations towards PARPis. In addition, the experimental results from constructing conditionally stable knock‐down cell lines suggest that knockdown of C1QBP significantly down‐regulates RAD51/54 expression (Figure S5A). More importantly, C1QBP over‐expression was able to counteract the inhibitory effect of PDBAG1 on HR‐related proteins such as BRCA1/2, RAD51 and RAD54 (Figure S5B).

### 2.9 PDBAG1 suppresses HR repair and induces PARPi sensitivity in vitro and in vivo

**FIGURE 8:**
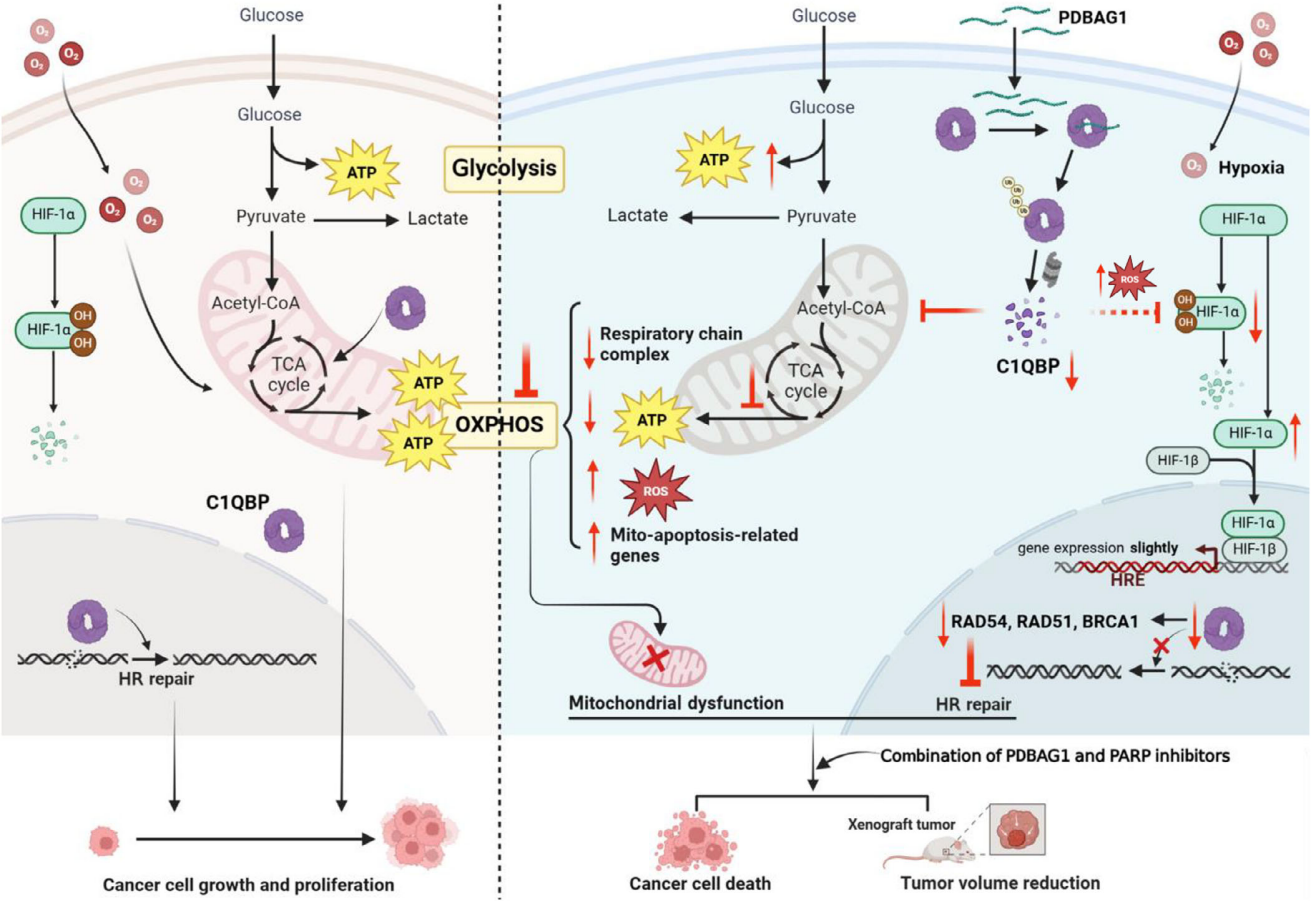
Schematic diagram of the mechanism of PDBAG1 acting on breast cancer cells (Created with BioRender.com).

To investigate whether enhanced PDBAG1‐induced impairment of DNA damage repair could potentiate the anti‐tumour effect of PARPi, we co‐treated TNBC cells with different concentrations of PDBAG1 and PARPi for 72 h in vitro to assess cell viability. We utilised two types of PARPis, olaparib and PJ34‐HCL, in combination with PDBAG1 to treat TNBC cells. Our findings revealed that the combination of PDBAG1 and olaparib did not further inhibit tumour cells; however, the PJ34‐HCL group exhibited significant effects, and its combined use with lower concentrations of PDBAG1 significantly enhanced TNBC inhibition (Figure 7E,F). Additionally, we investigated the expression of C1QBP in TNBC cells following treatment with varying concentrations of PARPis combined with PDBAG1 for 24 h, and observed no significant association between PARPi treatment and C1QBP protein expression (Figure S5C). To further validate the impact of PDBAG1 and PJ34‐HCL in vivo, we employed an orthotopic breast cancer tumourigenesis mouse model. MDA‐MB‐231 cells were transplanted into nude mouse mammary fat pads, and tumour volume as well as body weight measurements were taken every 3 days starting from day 7 post‐transplantation. From day 8 onwards, intraperitoneal injections were administered every 3 days using a dosage of 10 mg/kg for PDBAG1 and 1 mg/kg for PJ34‐HCL. The results demonstrated that PDBAG1 effectively inhibited TNBC growth in vivo; furthermore, combining it with PJ34‐HCL resulted in more pronounced effects on tumour volume (Figure 7G,H) as well as tumour weight (Figure 7I). After harvesting the tumour tissue, we employed immunohistochemistry (IHC) and western blot techniques to assess the expression of C1QBP protein in different treatment groups in vitro (Figure 7J,K). The findings indicated that there were no substantial changes observed in these two targets subsequent to the administration of PJ34‐HCL. However, a substantial reduction in C1QBP was observed in the treated groups containing PDBAG1. Furthermore, we evaluated the potential drug toxicity of PDBAG1 and PJ34‐HCL in vivo by examining HE staining of the spleen, liver and kidney as well as monitoring changes in body weight among nude mice across various treatment groups. Notably, no significant differences were detected regarding body weight fluctuations during or after drug administration (Figure S5D), while histological analysis through HE staining demonstrated no notable deviations from normal structure and morphology within the spleen, liver and kidney (Figure S5E). Moreover, the BRCA1‐mutant TNBC cell line HCC‐1937 could also be inhibited by PDBAG1 (Figure S5F). PDBAG1 significantly enhances the inhibitory effects of carboplatin on non‐BRCA mutant TNBC cells (Figure S5G) and still exerted significant anti‐tumour effects in immunocompetent mice (Figure S5H) (Figure 8).

## 3 DISCUSSION

In recent years, the prevalence of drug resistance in TNBC has posed a significant threat to patient outcomes. Despite ongoing research efforts that have identified new mechanisms and explored potential therapeutic targets,^30^, ^31^ there remains an urgent need for the discovery of novel treatment options. The majority of studies suggest that cancer progression is attributed to metabolic reprogramming, specifically the shift from OXPHOS to glycolysis or increased aerobic glycolysis.^8^, ^32^ However, accumulating evidence has revealed that tumour cells rely on both mitochondrial metabolism and aerobic glycolysis for their energy needs.^33^ Contrary to the prevailing notion of the Warburg effect, a recent study challenges this concept by demonstrating that various types of cancer, including TNBC,^34^ heavily depend on OXPHOS for survival.^35^ In fact, intracellular energy production primarily involves both glycolysis and mtOXPHOS. Consequently, targeting these bioenergetic pathways through inhibition of either glycolysis or OXPHOS has emerged as a potential therapeutic strategy in cancer treatment.^36^ Our results demonstrate that PDBAG1 disrupts mitochondrial function and suppresses OXPHOS by reducing C1QBP levels. This disruption leads to inhibited growth of TNBC cells observed in vitro as well as in a xenograft model. Consistently with our findings, Evans et al.^34^ reported that TNBC relies heavily on OXPHOS for survival and inhibiting this pathway may enhance the efficacy of targeted therapies. Overall, our findings contribute to advancing our understanding of tumour metabolism and provide a theoretical basis for targeted metabolic treatments in TNBC.

In fact, cancer cells can acquire a hybrid phenotype wherein both glycolysis and OXPHOS are utilised.^37^ This hybrid phenotype facilitates tumour cell survival in adverse environments, including treatment stress, and even promotes tumour metastasis and treatment resistance. We observed an increase in glycolysis when OXPHOS was suppressed by PDBAG1. Similar results showing decreased OXPHOS and increased reliance on glycolysis were also reported by other research groups.^38^, ^39^, ^40^ When mtOXPHOS metabolism or energy availability is disrupted, TNBC cells experience insufficient energy supply and are compelled to utilise glycolysis for energy production due to their metabolic flexibility. However, the glycolytic reserve capacity significantly decreased in TNBC cells exposed to PDBAG1 while the glycolytic respiratory capacity remained unchanged. The reduced glycolytic reserve and OXPHOS respiratory capacity reflect lower metabolic plasticity required to maintain intracellular ATP levels. Moreover, it suggests that PDBAG1 can exert energy stress on the tumour cells, thereby unveiling its inherent vulnerabilities.

C1QBP, targeted by PDBAG1, predominantly localises in the mitochondrial matrix and plays a crucial role in mitochondrial protein translation and function.^3^ It represents a promising therapeutic target for various human cancers, including breast cancer. We observed significant overexpression of C1QBP in the TNBC subtype, which correlated with worse DFS. Based on our findings, we believe PDBAG1 primarily affects tumour cells, with minimal toxicity to normal cells. Although PDBAG1's precursor peptide is derived from normal adipose tissue, its impact on adipose metabolism is likely less pronounced than in tumours. The effects of PDBAG1 on normal cells warrant further investigation. Previous studies have demonstrated that tumour homing peptide LyP‐1, VGF‐derived neuropeptide TLQP‐21, and tumour‐specific vascular homing peptide CGKRK can bind to cell surface‐associated C1QBP.^41^, ^42^, ^43^ M36 is a small molecule that binds C1QBP and inhibits its function, but it does not affect C1QBP protein levels in cells.^44^, ^45^ Since M36 binds to C1QBP in a competitive manner with LyP‐1,^44^ we speculate its mechanism of action may be similar to that of LyP‐1. In our study, we confirmed that the new peptide PDBAG1 also interacts directly with C1QBP in TNBC similar to previous findings. Importantly, to the best of our knowledge, PDBAG1 is the first peptide capable of effectively degrading C1QBP.

Although the data regarding the degradation of C1QBP may possess certain limitation, PDBAG1‐mediated down‐regulation of C1QBP protein has been observed in various tumour cell lines in this study. We hypothesise that PDBAG1 binding to C1QBP induces a conformational change, making C1QBP more prone to interaction with an E3 ubiquitin ligase and recognition for degradation via the ubiquitin–proteasome pathway. Although recent studies suggest Parkin may be an E3 ligase for C1QBP,^46^ earlier research indicates C1QBP is not a substrate for Parkin.^47^ Identifying the specific E3 ligase(s) involved in C1QBP ubiquitination will require further investigation, which we plan to explore in future studies. TPD has emerged as an innovative therapeutic approach for previously undruggable proteins.^48^ Recent research has demonstrated that the ubiquitin‐mediated degradation of key oncogenes can effectively impede tumour metastasis.^49^ Therefore, it is noteworthy that PDBAG1‐mediated degradation of C1QBP exhibits similarities to TPD strategies.

We also observed an increase in HIFs (HIF‐1α, HIF‐2α) and subsequent activation of the HIF signalling pathway expression following peptide treatment, which may directly contribute to the up‐regulation of glycolysis. In conjunction with our findings, we propose that mitochondrial dysfunction could resemble uncoupling at a specific point in the respiratory chain, where mitochondrial oxygen consumption persists but ATP production is impaired quantitatively. Previous studies have consistently associated hypoxia with invasive growth, metastasis, and poor prognosis for patients’ outcomes.^50^, ^51^ However, our research suggests that hypoxia is a multifaceted factor with complex implications on tumours; thus, its impact cannot be simply categorised as promoting or inhibiting cancer development. While enhanced glycolysis and elevated HIF‐1α expression have been reported to promote cancer cell survival,^52^, ^53^ we hypothesise that both enhanced glycolysis and up‐regulated HIF‐1α are necessary adaptive responses for cancer cells to survive after peptide PDBAG1 treatment‐induced mitochondrial functional impairment. Elevated HIF‐1α expression promotes enhanced glycolysis, a critical adaptive mechanism for cancer cells to generate ATP and support survival under these conditions. Furthermore, HIF‐1α up‐regulation could also trigger the pentose phosphate pathway,^54^ which helps mitigate ROS‐induced damage and provides additional cellular energy. Unfortunately, these coping strategies ultimately prove insufficient for their survival.

Under normal oxygen levels, the HIF‐1α protein undergoes degradation through the oxygen‐dependent proteasome system. However, under hypoxic conditions, this degradation process is inhibited, resulting in excessive accumulation of HIF‐1α. The MYC gene can activate mtOXPHOS, leading to the stabilisation of HIF‐1α through increased ROS production.^55^ The difference is that certain drugs that impair the electron transport chain in mitochondria can induce chemical hypoxia.^56^ Most related studies primarily focus on investigating the correlation between oxidative stress, ROS production, and hypoxia signalling pathways.^57^, ^58^ The mechanism of the hypoxia probe, such as pimonidazole, relies on competition for the first electron, allowing it to be activated in hypoxic conditions (O_2_ concentration below 1.3% or oxygen partial pressure ≤10 mm Hg).^25^ In our experiments conducted under normoxic conditions, it is unlikely that the intracellular oxygen partial pressure would drop dramatically. However, it is conceivable that a high concentration of reducing substances within the cells may interact with pimonidazole, resulting in what can be termed ‘normoxic hypoxia’. Recent research has demonstrated that DTT‐mediated reduction stress can activate intracellular hypoxia‐related signalling pathways even under normoxic conditions.^59^ According to the principle of hypoxy probe, we believe that after treated with PDBAG1 there is a significant presence of reducing substances within cells competing with O_2_ for binding to the oxygen probe. This competition often occurs due to the physiological reactions inside the cell under conditions of limited oxygen availability, leading to insufficient activation of signalling pathways associated with tumour cell oxygenation. Nevertheless, there is currently a scarcity of studies exploring the relationship between cellular reduction stress during normoxia and hypoxia, with an unclear underlying mechanism. The investigation of DNA damage repair inhibition induced by hypoxia is therefore crucial for the effective utilisation of its characteristics in subsequent research. Moreover, HIF‐1 consists of two subunits: HIF‐1β (constitutive and commonly stable) and HIF‐1α (inducible and flexibly regulated). It has been established that oxygen sensing depends on PHD,^60^, ^61^ FIH,^62^ complex III of the mitochondrial electron transport chain,^63^ ROS^64^ and histone demethylase KDM6A.^65^ These factors activate HIF transcription factors to induce a series of gene expressions that promote cellular adaptation to hypoxia for survival. In this study, we observed PDBAG1 stabilises HIF‐1α and HIF‐2α expression via an ROS‐dependent mechanism. Increased ROS levels after PDBAG1 treatment down‐regulate FIH and PHD2, reducing HIF hydroxylation and stabilising their expression. Consequently, although there was no significant promotion in the transcriptional activity of HIF‐1, only slight up‐regulation of target genes was observed. This finding is consistent with a down‐regulated glycolytic reserve which may explain why cells fail to survive despite increased expression of HIF‐1α. Therefore, we hypothesise that the reduction of C1QBP may cause mitochondrial damage, leading to compromised cellular function and the passive activation of HIF‐1α in tumour cells, ultimately enhancing glycolysis to compensate for the energy deficit.

Existing research has confirmed that hypoxia can disrupt DNA repair mechanisms through transcriptional and epigenetic regulation, resulting in genetic instability.^66^, ^67^ For example, the expression of HR repair‐related molecules decreases under hypoxic conditions.^68^ Additionally, studies have utilised the characteristic decline in DNA repair capabilities induced by hypoxia to investigate the effects of hypoxia inducers combined with PARPi on tumour cell death and enhanced anti‐cancer efficacy, achieving significant progress.^69^, ^70^ Moreover, recent studies have suggested that C1QBP may enhance HR repair by forming a complex with MRE11 and RAD50.^27^, ^71^ Consistent with this, down‐regulation of BRCA1/2 expression induced by PDBAG1 leads to HR repair deficiency, providing an opportunity for the use of PARPis in non‐BRCA mutant breast cancer treatment. In line with these findings, we also confirmed that disruption of C1QBP results in HR repair deficiency through the down‐regulation of BRCA1/2 and RAD51/54. Importantly, Glazer PM and his team conducted extensive experiments revealing altered DNA repair under hypoxic conditions, which could potentially be targeted therapeutically.^68^, ^70^, ^72^ Additionally, PDBAG1 triggers ROS production possibly due to mitochondrial damage. The dual nature of ROS has been well established as it can either promote or inhibit cancer incidence and progression. ROS act as anti‐tumourigenic species by inducing cellular senescence, apoptosis and DNA damage. Consistent with recent studies, we further validated an increase in ROS levels following PDBAG1 treatment. Mitochondria serve as the primary site for ROS generation and are also vulnerable to ROS attack. Damaged mitochondria release more ROS exacerbating the overall damage. Increased levels of ROS stabilise HIF‐1α leading to enhanced glycolysis as reported in recent studies.^73^, ^74^

Our findings demonstrate that the targeting of C1QBP by PDBAG1 leads to mitochondrial destruction, induction of homologous‐recombination deficiency, and sensitisation of TNBC cancer cells with non‐BRCA mutations to PARPis. The disruption and destruction of cellular metabolism homeostasis ultimately culminate in the demise of TNBC cancer cells and tumour regression. In summary, we have identified a novel peptide that targets C1QBP, which holds potential for enhancing sensitivity and synergising with other therapeutic molecules in the future. This discovery bears significant implications for precise targeted therapy of tumours.

## 4 MATERIALS AND METHODS

### 4.1 Sample collection

Three pairs of paratumour adipose tissue samples, obtained from patients with breast fibroma and breast cancer, were collected from Women's Hospital of Nanjing Medical University for peptide extraction and identification. The study was approved by the ethics committee of Women's Hospital of Nanjing Medical University, and informed consent was obtained from each patient through signed forms.

### 4.2 Peptide extraction and identification

The appropriate amount of adipose tissue samples was ground with liquid nitrogen and added to the protein lysate containing a protease inhibitor mixture (final concentrations: 1 mM PMSF, 2 mM EDTA, 10 mM DTT). The mixture was sonicated for 15 min on ice. Subsequently, the sample was centrifuged at 12 000 *g* for 30 min at 4°C and the supernatant was reserved. After measuring the protein concentration using the Bradford method, an equal amount of protein was ultrafiltered through a Vivacon 500 tube with a molecular weight cut‐off of 10 kDa (Sartorius) to collect the filtered liquid (by centrifuging at 12 000 *g* for 30 min at 4°C), thereby removing proteins and peptides larger than 10 kDa. The peptide samples were desalted using a Strata X C18 column (Phenomenex, Torrance, CA, USA) and subsequently lyophilised under vacuum. The dried peptide samples were reconstituted in mobile phase A (2% ACN, 0.1% FA) and separated using Thermo UltiMate3000 UHPLC system. The separation employed an effective gradient with a flow rate of 300 nL/min. The nanoliter liquid phase separation output directly connected to the mass spectrometer. The peptides eluted from liquid phase were ionised by the nanoESI source before entering into Orbitrap Fusion Lumos tandem mass spectrometer (Thermo Fisher Scientific, San Jose, CA) for data‐dependent acquisition (DDA) mode detection.

### 4.3 Cell culture and peptide preparations

The MDA‐MB‐231 and MDA‐MB‐468 cell lines were obtained from Shanghai Cell Bank (Shanghai, China) and cultured in DMEM medium (Gibco, USA) supplemented with 10% FBS at 37°C with 5% CO_2_ incubation conditions. All peptides used in this study were custom synthesised using standard Fmoc‐based solid‐phase peptide synthesis in collaboration with Shanghai Science Peptide Biological Technology Co (Supporting Information, Methods). The PDBAG1 sequence employed was GRKKRRQRRRPPQQASKKVCIVGSGNWGSAIA, which contained the cell‐penetrating sequence GRKKRRQRRRPPQQ. For the in vitro experiments, the peptides were dissolved in distilled water to a specific concentration before being directly added to the culture medium.

### 4.4 CCK‐8 assay, EdU assay and transwell assay

The cells were cultured at the corresponding cell densities in 96‐well plates. Following treatment with specific concentrations of reagents for designated durations, the CCK8 reagent was added to each well at a final ratio of 10% (v/v). After incubation for 2 h, the absorbance at 450 nm was measured using a microplate reader (H4, Synergy). For the Edu assay, logarithmic growth phase cells were inoculated into 96‐well plates at a density of 3 × 10^4^ cells/mL. Subsequently, they were treated with specific concentrations of reagents for designated durations and then replaced with EdU medium containing a final concentration of 10 µmol/L. The cells were incubated for an additional period of 2 h. Following PBS washing and fixation with 4% paraformaldehyde for 30 min, Apollo staining reaction solution was added according to instructions and allowed to react for another 30 min. This was followed by addition of DAPI staining reaction solution for a duration of 10 min before washing again with PBS. Three randomly selected fields of vision from each group in each well were calculated and photographed under a fluorescence microscope. For the transwell assay, transwell chambers (Millipore) with or without Matrigel (Sigma) were placed in a 24‐well plate with 8‐µm pores to evaluate invasion and migration, respectively. Cells were suspended in serum‐free medium containing a specific concentration of reagent, adjusted to a concentration of 3 × 10^4^ cells/mL, and separately seeded into the upper chamber for cell invasion and migration. Simultaneously, 850 µL of medium containing 10% FBS was added to the lower chamber. After a designated time period, the chambers were collected and stained for image observation and analysis. Three random fields of view were selected to quantify the number of cells that traversed through the microporous membrane.

### 4.5 Protein extraction, subcellular fractionation and Western blotting

The protein samples were extracted from the cells using Pierce™ IP lysis buffer (Thermo Scientific) supplemented with a cocktail of protease and phosphatase inhibitors (Roche). Subcellular fractionation was performed following the protocol described in the literature.^75^ Cytoplasmic isolation: A total of 2 × 10^8^ cells were digested, collected, washed with 1× PBS and then resuspended in 300 µL of pre‐chilled cytoplasmic lysis buffer containing a protease inhibitor cocktail. The cell suspension was incubated on ice for 5 min. Next, it was layered onto a sucrose solution layer at a volume ratio of 2.5:1 and centrifuged at 13 000  *g* for 10 min at 4°C. The supernatant obtained after centrifugation represented the cytoplasmic separation layer. Nuclear lysis: The nuclear pellet obtained from cytoplasmic isolation was washed with ice‐cold 1× PBS and then treated with an ice‐cold nuclear lysis buffer containing a protease inhibitor cocktail (100–200 µL). Gentle agitation was applied twice for 2 s each time followed by incubation on ice for another 2 min. Subsequently, centrifugation at 13 000 *g* for 2 min at 4°C allowed collection of the supernatant as the nuclear separation layer. Proteins were separated by SDS‐PAGE using various concentrations of resolving gels, transferred to PVDF membranes and subjected to immunoblotting analysis using specific primary antibodies (Table S1) overnight at 4°C followed by secondary antibody incubation at room temperature for 2 h.

### 4.6 Peptide pull‑down assay

The pull‐down experiments were conducted using biotin‐labelled peptides. Following six washes with PBS buffer, streptavidin magnetic beads were incubated overnight at 4°C with 2 µg of biotin‐labelled peptides. MDA‐MB‐231 and MDA‐MB‐468 cells were collected and lysed in IP lysis buffer supplemented with a cocktail of protease inhibitors. Subsequently, the streptavidin magnetic beads bound to the peptides were incubated overnight at 4°C. The supernatant was removed by placing the samples on a magnet rack, followed by six washes with IP lysis buffer supplemented with a cocktail of protease inhibitors. The captured proteins were detected through silver staining or western blot analysis.

### 4.7 Total cell protein co‐IP

Take 0.8–1.0 mg of the collected total cell protein and add 2 µL of the specific antibody. Invert and mix the solution, place it on a rotary mixer, and incubate at 4°C overnight. On the following day, take 20 µL of A/G magnetic beads and wash them with cell lysate for four to six times. Then, add the antibody‐protein complexes that were incubated overnight to the magnetic beads. Incubated by inverting and mixing for 2 h at room temperature, followed by placing them on a magnetic rack for 2 min to separate supernatant from beads. Discard the supernatant and repeat this washing step four times using cell lysate each time before placing them on a magnetic rack for another 2 min each time to remove any remaining supernatant completely. Finally, add 30 µL of 1× protein loading buffer to each sample, thoroughly mix through mediation, and heat in boiling water bath for 5–7 min. After standing on a magnetic stand for an additional 2 min, they can be used for subsequent western blotting experiments.

### 4.8 SEC and ITC assay

The human C1QBP_74–282_ *E. coli* expression vector was a gift from Dr Adrian R. Krainer, Cold Spring Harbor Laboratory, USA. Expression and purification of C1QBP were performed following the protocol described previously.^76^ SEC was conducted using Superdex 200 Increase 10/300 GL column (GE healthcare) in a buffer containing 20 mM HEPES at pH 7.4 and 140 mM NaCl. ITC experiments were carried out to investigate the binding of PDBAG1 (500 µM, prepared in ITC buffer) to C1QBP_74–282_ (17.0 µM, prepared in a buffer consisting of 20 mM HEPES at pH 7.5, 600 mM NaCl, 1 mM EDTA, 1 mM EGTA, and 5% glycerol). The ITC measurements were performed using a MicroCal VP ITC system (Malvern) at a temperature of 25°C. The ITC cell contained a volume of 250 µL C1QBP_74–282_ while the syringe was loaded with 80 µL PDBAG1 solution (reference power: 5 μcal/s; syringe stirring speed: 750 rpm). A series of injections including an initial injection of 0.4 µL followed by 19 × 2µL injections were made during the experiment. The obtained data were fitted to a one set‐of‐sites binding model.

### 4.9 Molecular docking simulations

Molecular docking simulations were conducted using the online prediction tool H‐dock^77^ (http://hdock.phys.hust.edu.cn/). The protein structure data of C1QBP were obtained from the PDB database (crystal structure of human p32, code: 1p32). The structures of PDBAG1 were simulated based on the H‐dock database. The docking model with a higher score was selected.

### 4.10 ROS and cell apoptosis analysis of cells

The MDA‐MB‐231 and MDA‐MB‐468 cells were harvested after treatment with a series of concentrations of PDBAG1 using EDTA‐free trypsin for the analysis of ROS and cell apoptosis. For the ROS assay, the cells were suspended in serum‐free medium containing 10 µM DCFH‐DA for 60 min. For the apoptosis assay, the cells were suspended in 500 µL binding buffer mixed with 10 µL Annexin V/PI (BD Bioscience, USA) at room temperature for 10 min. The stained cells were washed twice with serum‐free medium (for ROS) or PBS buffer (for apoptosis) before flow cytometry analysis. All samples were analysed using flow cytometry software Kaluza (Beckman Coulter), following the manufacturer's instructions for data acquisition where 10 000 events per sample were collected.

### 4.11 FITC‐labelled PDBAG1 distribution test

The cells were seeded in a 24‐well plate, and FITC‐labelled PDBAG1 was added to the cell culture medium at a final concentration of 50 µM. After three washes with PBS, performed 6 h post peptide addition, the samples were fixed with 4% paraformaldehyde for 15 min and stained with DAPI (5 µg/mL) for 5 min. Following PBS washing, fluorescence microscopy was employed for observation.

### 4.12 RNA‐seq analysis and RT‐qPCR

Total RNA was extracted from MDA‐MB‐231 cells treated with either vehicle or PDBAG1 using TRIzol reagent (Thermo Fisher Scientific). The concentration of RNA was determined by measuring the absorbance ratio at 260/280 nm using a spectrophotometer (NanoDrop ND‐1000 Thermo Scientific). Purified total RNA was sent to BGI (Shenzhen, China) for mRNA library construction and sequencing on the BGISEQ500 platform. The resulting RNA‐seq data were analysed using the BGI bioinformatics platform. HISAT2 was used to map clean reads to the human genome (GRCh38.p13), and gene expression analysis involved counting matched reads that were normalised to FPKM values. Genes exhibiting changes in expression greater than 1.5‐fold with *q* < 0.05 were selected as target genes, and Hallmarks were analysed via GSEA and gene network analysis. KEGG pathway analysis using R package facilitated heat map generation, clustering, data mining, graphic presentation processes, among others. Data analyses were performed online through Dr Tom system customised for internal use at BGI; RT‐qPCR experiments employed HiScript III RT SuperMix for qPCR and ChamQ Universal SYBR qPCR Master Mix kit according to manufacturer's instructions; primer sequences are listed in Table S3.

### 4.13 siRNA and plasmid transfection

(1) *siRNA transfection*: Cells at approximately 70% confluency can be used for transfection. The preparation process of the transfection reagent is as follows (taking cell transfection in a six‐well plate as an example): Take 3 µL of siRNA and 5 µL of Lipo3000, respectively, and add 125 µL of Opti‐MEM to each. Gently mix and let it stand for 5 min. Transfer the liquid from one tube to another, gently mix again and let it stand for 15 min. Drop the prepared transfection reagent into the corresponding wells while gently shaking to ensure thorough mixing. After incubating for 48 h, cells can be collected for subsequent experiments (Si‐RNA sequence listed in Table S2). (2) *Plasmid transfection*: Cells at approximately 70% confluency can be used for transfection. The preparation process of the transfection reagent is as follows (taking cell transfection in a six‐well plate as an example): Add 2 µg plasmid to100 µL Opti‐MEM and mix gently; then add 3 µL Jetprime transfection reagent and mix gently. Let it stand for 20 min. The subsequent transfection process is the same as siRNA transfection.

### 4.14 Cell metabolism assay

Metabolic assays were conducted on TNBC cells using the Seahorse instrument (Agilent, USA) with the glycolytic stress assay kit and mito‐stress assay kit. 80 000 cells were seeded in XF24‐well plates, treated with PDBAG1 for 24 h, and processed according to the manufacturer's instructions. The intracellular ATP levels were quantified using an ATP sensor (MaLionR plasmid). The MaLionR plasmid was a gift from Tetsuya Kitaguchi (Addgene plasmid # 113908; http://n2t.net/addgene:113908; RRID: Addgene_113908). Intracellular ATP was measured using an ATP detection kit (Dojindo, Japan). 3000 cells were seeded in a 96‐well plate and after treatment with PDBAG1 at corresponding concentrations for 24 h, ATP content was detected by utilising appropriate inhibitors and detection reagents as per instructions.

### 4.15 Immunofluorescence and hypoxyprobe assay

The Nunc™ Lab‐Tek™ II Chamber Slide™ (154534PK; Thermo Scientific) was inoculated with 3000 cells and treated with various concentrations of reagents for 24 h. Subsequently, the medium was replaced with medium containing either 0.5 mg/mL mitotracker deep red or 200 µM hypoxyprobe™‐1 (Hypoxyprobe, Inc). The samples were then incubated at 37°C in a CO_2_ incubator for 60 min before removing the medium. Fixation of the samples was achieved by treating them with 4% poly formaldehyde at room temperature for 15 min, followed by membrane permeabilisation using TritonX‐100 PBS solution (0.2%) for an additional 5 min. Blocking of the membrane was performed using goat serum at room temperature for 1 h. Next, primary antibody incubation (C1QBP, HIF‐1α, FITC‐MAb1 clone:4.3.11.3) took place overnight at 4°C, followed by secondary antibody incubation (Alexa Fluor 488 from Thermo Fisher) for 1 h at room temperature. Hoechst33342 dye (2 µg/mL from Thermo Scientific) was used to stain nuclei and allowed to react at room temperature for 5 min before observation under a confocal microscope.

### 4.16 C1QBP overexpression and pTRIPZ shRNAmir lentivirus packaging and cell line construction

In collaboration with Shanghai Genepharma company, we generated the C1QBP overexpression lentivirus package plasmid in conjunction with the pTRIPZ‐plasmid harbouring sh‐C1 and sh‐C2 sequences. The steps involved in lentivirus packaging were as follows: when HEK‐293T cells reached 80–90% confluency in a 10 cm dish, they were co‐transfected with pTRIPZ (10 µg), PAX2 (7.5 µg) and PMD2.g (5 µg). After 18–20 h, the medium was replaced with 20 mL of complete medium, and another collection of complete medium was performed after an additional 48 h to obtain virus particles. For cell line construction, MDA‐MB‐231 cells were seeded into six‐well plates. Once reaching a density of 50%, the culture supernatant containing virus particles (1 mL) was added followed by a medium change after 24 h. Subsequently, complete medium supplemented with puromycin at a concentration of 2 µg/mL was used for selection after an additional incubation period of 48 h. The selected cells were maintained for at least three successive passages to ensure stable expression screening completion. RFP expression was observed following induction with DOX at a concentration of 2 µg/mL for 48 h, while protein expression was assessed using western blot analysis.

### 4.17 Animal studies

Four‐week‐old BALB/c nude mice (Charles River Laboratories, Beijing, China) were subcutaneously inoculated in the breast fat pad with 1 × 10^7^ MDA‐MB‐231 cells. The treatment group received 10 mg/kg PDBAG1, while the control group was administered an equal volume of PBS. For preliminary experiment of immunocompetent mice, 4‐week‐old BALB/c mice (Charles River Laboratories) were subcutaneously inoculated in the mammary fat pad with 1 × 10^6^ 4T1 cells. The treatment group received 10 mg/kg PDBAG1, while the control group was administered an equal volume of PBS. For evaluating the PJ34‐HCL combination, 28 randomly assigned BALB/c nude mice were divided into respective treatment groups as follows: Group 1 served as a negative control and received PBS; Group 2 received intraperitoneal injections of 10 mg/kg PDBAG1 every 3 days; Group 3 received 1 mg/kg PJ34‐HCL every 3 days; Group 4 was treated with a combination of 10 mg/kg PDBAG1 and 1 mg/kg PJ34‐HCL every 3 days. Both PDBAG1 and PJ34‐HCL were dissolved in specific concentrations of normal saline and administered intraperitoneally. All mice were sacrificed 4 weeks after inoculation, and their subcutaneous xenografts were resected and weighed. Subsequently, formalin‐fixed subcutaneous tumours were processed into paraffin‐embedded sections for routine IHC analysis. The murine xenograft assay was approved by the Institutional Ethics Committee of Nanjing Medical University (Nanjing, China). Immunohistochemical staining using anti‐C1QBP antibodies was performed on tumour sections from the mice to determine their levels. After blocking, the tumour sections were incubated overnight with primary antibodies followed by secondary antibody incubation before being treated with diaminobenzidine and counterstained with haematoxylin. All tissues were observed under a microscope (Carl Zeiss, Oberkochen, Germany) at a magnification of ×40.

### 4.18 Statistical analysis

The quantitative data were expressed as means ± standard deviation (SD). Statistical comparisons were performed using Student's *t*‐test and ANOVA test. All statistical analyses were conducted using PRISM6 software (Dotmatics, England). A *p* value of less than 0.05 was considered statistically significant.

## AUTHOR CONTRIBUTIONS

Conceptualisation was performed by Xingxing Li, Mingming Lv and Cheng Lu. Visualisation was performed by Xingxing Li, Yue Wu and Min Zhang. Methodology was designed by Xingxing Li, Yue Wu, Min Zhang, Fengliang Wang and Hong Yin. Formal analysis was performed by Xingxing Li, Yue Wu, Yanrong Zhang, Shuli Zhao and Jiehua Ma. Investigation was performed by Xingxing Li and Yue Wu. Writing—original draft was completed by Xingxing Li and Mingming Lv. Reviewing and editing the original draft was completed by Xingxing Li, Yue Wu and Mingming Lv. Project administration and supervision were performed by Xingxing Li, Mingming Lv and Cheng Lu.

## CONFLICT OF INTEREST STATEMENT

The authors have no conflicts of interest to declare.

## ETHICS STATEMENT

The authors have nothing to report.

## Supporting information



Supporting Information

## Data Availability

The complete dataset can be found in either the main text or the supplemental information. The raw data have been deposited in the GEO database under accession code GSE205489.
