# Supplementary material for: A new peptide inhibitor of C1QBP exhibits potent anti‐tumour activity against triple negative breast cancer by impairing mitochondrial function and suppressing homologous recombination repair
Source: Clin Transl Med. 2025 Jan 2;15(1):e70162. doi: 10.1002/ctm2.70162 (PMC11695203; doi:10.1002/ctm2.70162)
Supplement: Supplementary file 1 — Supporting Information [file CTM2-15-e70162-s001.docx]

**Table S1. Antibodies used in the study**

| Antibody | Catalog | Supplier | Isotype/Source |
| --- | --- | --- | --- |
| HIF-1α (D1S7W) XP® Rabbit mAb  Hydroxy-HIF-1α (Pro564) Rabbit mAb  HIF-1β/ARNT (D28F3) XP® Rabbit mAb  HIF-2α (D6T8V) Rabbit mAb  FIH (D19B3) Rabbit mAb  PHD-2/Egln1 (D31E11) Rabbit mAb  VHL Antibody  ATM (D2E2) Rabbit mAb  Phospho-ATM (Ser1981) (D25E5) Rabbit mAb  Rad51 (D4B10) Rabbit mAb  BRCA1 (A8X9F) Rabbit mAb  BRCA2 (D9S6V) Rabbit mAb  Rad54 (D4W3Z) Rabbit mAb  p95/NBS1 (D6J5I) Rabbit mAb  Phospho-p95/NBS1 (Ser343) Antibody  CtIP (D76F7) Rabbit mAb  HA-Tag (C29F4) Rabbit mAb  β-Actin (13E5) Rabbit mAb  AMPKα (D5A2) Rabbit mAb  Phospho-AMPKα (Thr172) (40H9) Rabbit mAb  Anti-rabbit IgG, HRP-linked Antibody | 36169  3434  5537  59973  4426  4835  68547  2873  13050  8875  14823  10741  15016  14956  3001  9201  3724  4970  5831  2535  7074 | Cell Signaling Technology | Rabbit IgG Rabbit IgG Rabbit IgG Rabbit IgG Rabbit IgG Rabbit IgG Rabbit  Rabbit IgG Rabbit IgG Rabbit IgG Rabbit IgG Rabbit IgG Rabbit IgG Rabbit IgG Rabbit Rabbit IgG  Rabbit  Rabbit  Rabbit IgG  Rabbit IgG  Goat |
| DYKDDDDK tag Polyclonal antibody (Binds to FLAG® tag epitope)  DHX57 Polyclonal antibody  MYBBP1A Polyclonal antibody  TCOF1 Polyclonal antibody  SLC25A22 Polyclonal antibody  C1QBP Polyclonal antibody  ATP5A1 Polyclonal antibody | 20543-1-AP  24525-1-AP  14524-1-AP  11003-1-AP  25402-1-AP  24474-1-AP  14676-1-AP | Proteintech | Rabbit  Rabbit  Rabbit Rabbit Rabbit Rabbit Rabbit |
| Anti-GC1qR Polyclonal antibody (For co-IP)  Total OXPHOS Rodent WB antibody Cocktail (samples heated to 50℃) | ab101267  ab1110413 | Abcam | Rabbit  Mouse |

**Table S2. Si-RNA sequence used in this study**

| Si-RNA | Target-sequence |
| --- | --- |
| Si-C1QBP-1 | GGACAACACTTTTGCAGAT |
| Si-C1QBP-2 | GAGTCTGAATGGAAGGATA |
| Si-C1QBP-3 | GTTGGACTGTCATTATCCA |

**Table S3.** **Real-time PCR primers used in this study**

| Primer | 5’ to 3’ |
| --- | --- |
| HK1-F | CACATGGAGTCCGAGGTTTATG |
| HK1-R | CGTGAATCCCACAGGTAACTTC |
| PDK1-F | CTGTGATACGGATCAGAAACCG |
| PDK1-R | TCCACCAAACAATAAAGAGTGCT |
| PDK3-F | CGCTCTCCATCAAACAATTCCT |
| PDK3-R | CCACTGAAGGGCGGTTAAGTA |
| PGM1-F | GATGGGGATCGAAACATGATTCT |
| PGM1-R | GCTGGAAATACGGAATGCTGAA |
| GYS1-F | CAGCGCGGACCAACAATTTC |
| GYS1-R | TCCTCCCGAACTTTTCCTTCA |
| GBE1-F | CAAAGTATGTGGTTCGTGAAGGT |
| GBE1-R | GATTGCCATCAACTGAATGCAG |
| Actin-F | GCGAGAAGATGACCCAGATC |
| Actin-R | GGATAGCACAGCCTGGATAG |
| PKM2-F | ATAACGCCTACATGGAAAAGTGT |
| PKM2-R | TAAGCCCATCATCCACGTAGA |
| PGK1-F | GAACAAGGTTAAAGCCGAGCC |
| PGK1-R | GTGGCAGATTGACTCCTACCA |
| C1QBP-F | TGATGGTGAGGAGGAACCCT |
| C1QBP-R | CGCCAGTGGACTGAAAGCTA |
| BRCA1-F | GCCAAGGCAAGATCTAGAGG |
| BRCA1-R | GTTGCCAACACGAGCTGA |
| BRCA2-F | GTGAAGAGCAGTTAAGAG |
| BRCA2-R | GACATCCCTTGATAAACC |

**Table S4. 109 proteins identified as interacting with PDBAG1.**

| **Protein_ID** | **Protein_Qscore (PDBAG1-1)** | **Protein_Qscore (PDBAG1-2)** | **Protein_Qscore (sum)** | **iBAQ (PDBAG1-1)** | **iBAQ (PDBAG1-2)** | **iBAQ (sum)** |
| --- | --- | --- | --- | --- | --- | --- |
| sp\|Q9H936\|GHC1_HUMAN | 9.094861245 | 10.80425606 | 19.8991173 | 1537260.06 | 39812.1396 | 1577072.2 |
| sp\|P61981\|1433G_HUMAN | 6.119341349 | 6.512114028 | 12.63145538 | 621984.952 | 634560.645 | 1256545.6 |
| sp\|Q07021\|C1QBP_HUMAN | 16.38373363 | 21.7324324 | 38.11616602 | 857279.802 | 35360.3696 | 892640.171 |
| sp\|Q86VM9\|ZCH18_HUMAN | 46.55356934 | 82.04140352 | 128.5949729 | 151073.803 | 664693.39 | 815767.193 |
| sp\|P55201\|BRPF1_HUMAN | 62.57031069 | 75.22189198 | 137.7922027 | 196770.342 | 329309.889 | 526080.231 |
| sp\|Q9H307\|PININ_HUMAN | 36.88050395 | 65.15309323 | 102.0335972 | 84488.9281 | 172918.136 | 257407.064 |
| sp\|Q6P158\|DHX57_HUMAN | 78.1146662 | 76.24881305 | 154.3634792 | 113311.639 | 119469.292 | 232780.931 |
| sp\|Q9BQG0\|MBB1A_HUMAN | 85.48732176 | 100.1968574 | 185.6841791 | 108759.607 | 85839.2659 | 194598.873 |
| sp\|Q8N9T8\|KRI1_HUMAN | 7.828268434 | 6.726841111 | 14.55510955 | 182622.226 | 2503.4401 | 185125.666 |
| sp\|P61619\|S61A1_HUMAN | 5.784793191 | 13.18550851 | 18.9703017 | 40368.7759 | 130026.566 | 170395.342 |
| sp\|Q9NPF4\|OSGEP_HUMAN | 2.447351236 | 8.764432764 | 11.211784 | 740.188001 | 123003.077 | 123743.265 |
| sp\|O95983\|MBD3_HUMAN | 2.273432515 | 2.136888913 | 4.410321428 | 0 | 118757.794 | 118757.794 |
| sp\|Q9NR50\|EI2BG_HUMAN | 2.695496525 | 2.542326796 | 5.237823322 | 54568.5109 | 55114.6633 | 109683.174 |
| sp\|Q13428\|TCOF_HUMAN | 35.48576942 | 77.69272153 | 113.1784909 | 46398.0355 | 55808.9977 | 102207.033 |
| sp\|Q9Y625\|GPC6_HUMAN | 7.757359513 | 10.45864469 | 18.2160042 | 25262.4938 | 71173.3495 | 96435.8433 |
| sp\|Q99613\|EIF3C_HUMAN | 13.01268017 | 39.50802624 | 52.52070641 | 7610.64724 | 79577.6325 | 87188.2797 |
| sp\|Q86SQ0\|PHLB2_HUMAN | 28.93447902 | 54.46711061 | 83.40158963 | 9095.78324 | 66636.9529 | 75732.7362 |
| sp\|Q7L523\|RRAGA_HUMAN | 15.18019681 | 13.7183767 | 28.89857351 | 21637.6146 | 52615.8398 | 74253.4544 |
| sp\|O14646\|CHD1_HUMAN | 34.6999201 | 35.27608551 | 69.97600561 | 58628.5347 | 10854.1936 | 69482.7282 |
| sp\|Q96JK2\|DCAF5_HUMAN | 35.63508433 | 48.03594211 | 83.67102644 | 31458.1755 | 32951.8628 | 64410.0384 |
| sp\|Q52LW3\|RHG29_HUMAN | 34.89264703 | 75.49568534 | 110.3883324 | 9781.25847 | 48307.9261 | 58089.1845 |
| sp\|Q9UHB7\|AFF4_HUMAN | 25.03494163 | 50.98289813 | 76.01783976 | 22264.244 | 32689.2152 | 54953.4592 |
| sp\|P09651\|ROA1_HUMAN | 11.70911375 | 12.22431642 | 23.93343018 | 13753.5901 | 38350.6507 | 52104.2408 |
| sp\|Q9Y5B9\|SP16H_HUMAN | 22.53401308 | 43.96837354 | 66.50238662 | 24103.1428 | 22815.6266 | 46918.7695 |
| sp\|P17655\|CAN2_HUMAN | 18.54342611 | 22.46293836 | 41.00636447 | 23037.7394 | 23754.8813 | 46792.6208 |
| sp\|Q6IPU0\|CENPP_HUMAN | 4.095933406 | 2.761702631 | 6.857636037 | 8519.64021 | 34298.9413 | 42818.5815 |
| sp\|Q9UQ35\|SRRM2_HUMAN | 22.65850918 | 58.65801899 | 81.31652817 | 12229.1214 | 30456.2431 | 42685.3645 |
| sp\|Q96RD6\|PANX2_HUMAN | 2.777338118 | 2.165627548 | 4.942965666 | 19341.7488 | 18554.82 | 37896.5688 |
| sp\|Q8N335\|GPD1L_HUMAN | 8.586759886 | 4.34648648 | 12.93324637 | 34673.7523 | 2779.34902 | 37453.1013 |
| sp\|Q9Y673\|ALG5_HUMAN | 4.855189014 | 12.2992492 | 17.15443822 | 2848.99085 | 31120.0838 | 33969.0746 |
| sp\|Q9UKV3\|ACINU_HUMAN | 2.610415758 | 14.27168337 | 16.88209913 | 454.094082 | 32188.6133 | 32642.7074 |
| sp\|Q5T749\|KPRP_HUMAN | 2.202683277 | 2.761702631 | 4.964385908 | 0 | 32369.7219 | 32369.7219 |
| sp\|Q2NKX9\|CB068_HUMAN | 4.095933406 | 2.542326796 | 6.638260203 | 15114.931 | 17239.7796 | 32354.7106 |
| sp\|Q14978\|NOLC1_HUMAN | 23.09949389 | 26.21368919 | 49.31318308 | 14685.7006 | 17585.3073 | 32271.0078 |
| sp\|P01857\|IGHG1_HUMAN | 5.095079872 | 2.043843522 | 7.138923394 | 27490.3388 | 4584.00805 | 32074.3468 |
| sp\|P62136\|PP1A_HUMAN | 2.4872289 | 3.305093795 | 5.792322695 | 19732.4438 | 8560.1784 | 28292.6222 |
| sp\|Q9NRR4\|RNC_HUMAN | 20.42854809 | 36.75438699 | 57.18293508 | 9087.31661 | 17948.999 | 27036.3156 |
| sp\|O00203\|AP3B1_HUMAN | 11.04201719 | 14.5453307 | 25.58734789 | 23203.555 | 653.584763 | 23857.1398 |
| sp\|Q9NVZ3\|NECP2_HUMAN | 2.202683277 | 3.305093795 | 5.507777071 | 22438.05 | 1407.55138 | 23845.6013 |
| sp\|P56537\|IF6_HUMAN | 2.695496525 | 2.393451915 | 5.08894844 | 23248.0024 | 0 | 23248.0024 |
| sp\|Q15366\|PCBP2_HUMAN | 5.224689355 | 3.305093795 | 8.529783149 | 18211.1057 | 4317.22959 | 22528.3353 |
| sp\|Q9HAB8\|PPCS_HUMAN | 4.095933406 | 11.63958081 | 15.73551422 | 984.354167 | 17553.7407 | 18538.0949 |
| sp\|Q96ST3\|SIN3A_HUMAN | 2.4872289 | 12.49138456 | 14.97861346 | 9787.85222 | 7231.4881 | 17019.3403 |
| sp\|Q6ZNA4\|RN111_HUMAN | 12.19329859 | 16.34455323 | 28.53785182 | 7923.82371 | 8273.02397 | 16196.8477 |
| sp\|Q96GD0\|PLPP_HUMAN | 11.08426341 | 10.80425606 | 21.88851947 | 12091.447 | 3634.77218 | 15726.2191 |
| sp\|P43897\|EFTS_HUMAN | 4.355697048 | 9.488543742 | 13.84424079 | 2357.06661 | 12984.2903 | 15341.3569 |
| sp\|Q16610\|ECM1_HUMAN | 2.695496525 | 3.062829683 | 5.758326208 | 9951.55224 | 4091.48907 | 14043.0413 |
| sp\|P11274\|BCR_HUMAN | 19.701656 | 30.01465618 | 49.71631218 | 7653.80929 | 5710.32002 | 13364.1293 |
| sp\|O60524\|NEMF_HUMAN | 14.34253195 | 17.81904128 | 32.16157323 | 6554.42872 | 6459.77235 | 13014.2011 |
| sp\|Q14151\|SAFB2_HUMAN | 7.944485679 | 8.99054222 | 16.9350279 | 0 | 12510.6177 | 12510.6177 |
| sp\|P50991\|TCPD_HUMAN | 4.230818556 | 2.334141337 | 6.564959892 | 2967.58775 | 8195.22553 | 11162.8133 |
| sp\|P0C2W1\|FBSP1_HUMAN | 2.695496525 | 2.761702631 | 5.457199157 | 8563.2899 | 2379.29217 | 10942.5821 |
| sp\|Q9H4L7\|SMRCD_HUMAN | 6.873271525 | 11.99806675 | 18.87133828 | 6330.88593 | 3373.30771 | 9704.19364 |
| sp\|O43933\|PEX1_HUMAN | 2.028135279 | 5.225512839 | 7.253648118 | 58.1821168 | 8424.56719 | 8482.7493 |
| sp\|O95819\|M4K4_HUMAN | 13.29987508 | 28.24675698 | 41.54663206 | 2356.14628 | 6055.1856 | 8411.33189 |
| sp\|Q9UHQ9\|NB5R1_HUMAN | 4.095933406 | 3.062829683 | 7.158763089 | 1418.33016 | 6833.45793 | 8251.78809 |
| sp\|P17480\|UBF1_HUMAN | 5.050770633 | 4.668282674 | 9.719053307 | 6262.42168 | 1877.0513 | 8139.47299 |
| sp\|Q14152\|EIF3A_HUMAN | 2.777338118 | 12.66048697 | 15.43782509 | 532.525661 | 7582.81051 | 8115.33617 |
| sp\|Q9NRY6\|PLS3_HUMAN | 4.095933406 | 2.380354631 | 6.476288038 | 922.178956 | 6231.08342 | 7153.26237 |
| sp\|Q13505\|MTX1_HUMAN | 2.777338118 | 2.228834438 | 5.006172556 | 1662.87332 | 5185.04396 | 6847.91728 |
| sp\|Q9UFF9\|CNOT8_HUMAN | 2.447351236 | 6.610187589 | 9.057538825 | 0 | 6787.68116 | 6787.68116 |
| sp\|O60671\|RAD1_HUMAN | 2.4872289 | 5.808789593 | 8.296018494 | 2662.08994 | 3970.09428 | 6632.18422 |
| sp\|P26640\|SYVC_HUMAN | 4.530704144 | 11.75224485 | 16.282949 | 3065.22576 | 3541.06908 | 6606.29484 |
| sp\|P61313\|RL15_HUMAN | 8.352529098 | 3.305093795 | 11.65762289 | 3980.09798 | 2205.73144 | 6185.82942 |
| sp\|Q9Y6C9\|MTCH2_HUMAN | 2.202683277 | 4.885522262 | 7.088205538 | 3837.60783 | 1990.38158 | 5827.9894 |
| sp\|P11908\|PRPS2_HUMAN | 4.095933406 | 2.761702631 | 6.857636037 | 4664.3313 | 1096.88015 | 5761.21145 |
| sp\|Q5JTH9\|RRP12_HUMAN | 10.29622755 | 15.62692973 | 25.92315727 | 3600.41507 | 1401.95201 | 5002.36708 |
| sp\|Q8WYH8\|ING5_HUMAN | 2.243443753 | 2.165627548 | 4.409071301 | 4868.05214 | 0 | 4868.05214 |
| sp\|Q8NI27\|THOC2_HUMAN | 8.562131309 | 15.79942792 | 24.36155923 | 1477.18313 | 3299.37445 | 4776.55758 |
| sp\|Q00839\|HNRPU_HUMAN | 6.298616683 | 4.608125262 | 10.90674195 | 2457.42722 | 2141.06965 | 4598.49686 |
| sp\|Q8N122\|RPTOR_HUMAN | 2.303431313 | 14.5992793 | 16.90271061 | 0 | 4542.80563 | 4542.80563 |
| sp\|P04040\|CATA_HUMAN | 2.892396595 | 6.125659365 | 9.018055961 | 0 | 4083.81975 | 4083.81975 |
| sp\|O96028\|NSD2_HUMAN | 2.777338118 | 10.3803598 | 13.15769791 | 0 | 3672.50082 | 3672.50082 |
| sp\|Q92922\|SMRC1_HUMAN | 8.642433842 | 20.26755674 | 28.90999058 | 360.064632 | 3083.4972 | 3443.56183 |
| sp\|Q8N0Z8\|PUSL1_HUMAN | 6.791429932 | 7.108189111 | 13.89961904 | 2599.79676 | 440.520372 | 3040.31713 |
| sp\|Q5SSJ5\|HP1B3_HUMAN | 2.043475244 | 6.066796426 | 8.110271669 | 0 | 2966.68154 | 2966.68154 |
| sp\|P56937\|DHB7_HUMAN | 4.095933406 | 4.34648648 | 8.442419886 | 2962.84968 | 0 | 2962.84968 |
| sp\|Q7Z7A3\|CTU1_HUMAN | 2.023407943 | 3.305093795 | 5.328501737 | 1664.3477 | 1111.79635 | 2776.14405 |
| sp\|P49411\|EFTU_HUMAN | 4.095933406 | 2.761702631 | 6.857636037 | 0 | 2528.64052 | 2528.64052 |
| sp\|Q9Y2X3\|NOP58_HUMAN | 2.023407943 | 4.34648648 | 6.369894423 | 0 | 2390.88447 | 2390.88447 |
| sp\|Q96GD4\|AURKB_HUMAN | 2.202683277 | 5.457775297 | 7.660458573 | 0 | 2327.04599 | 2327.04599 |
| sp\|Q9NXK8\|FXL12_HUMAN | 4.095933406 | 8.980685424 | 13.07661883 | 2211.006 | 0 | 2211.006 |
| sp\|P07477\|TRY1_HUMAN | 2.892396595 | 3.305093795 | 6.19749039 | 0 | 1900.16903 | 1900.16903 |
| sp\|P15927\|RFA2_HUMAN | 2.023407943 | 6.45776958 | 8.481177523 | 0 | 1880.42491 | 1880.42491 |
| sp\|Q96T58\|MINT_HUMAN | 4.095933406 | 3.305093795 | 7.401027201 | 0 | 1784.93487 | 1784.93487 |
| sp\|Q13144\|EI2BE_HUMAN | 2.008388544 | 2.334141337 | 4.342529881 | 0 | 1765.32206 | 1765.32206 |
| sp\|P11216\|PYGB_HUMAN | 2.892396595 | 8.44929918 | 11.34169578 | 0 | 1725.2616 | 1725.2616 |
| sp\|Q96PV7\|F193B_HUMAN | 2.202683277 | 6.367923477 | 8.570606754 | 448.403204 | 1147.25443 | 1595.65764 |
| sp\|Q6PKG0\|LARP1_HUMAN | 2.354521718 | 7.260375479 | 9.614897197 | 334.498371 | 1058.56456 | 1393.06294 |
| sp\|O75151\|PHF2_HUMAN | 8.191866813 | 4.34648648 | 12.53835329 | 1140.11205 | 0 | 1140.11205 |
| sp\|Q8WVM7\|STAG1_HUMAN | 2.542688948 | 3.305093795 | 5.847782742 | 518.045308 | 207.482233 | 725.52754 |
| sp\|Q9NWS8\|RMND1_HUMAN | 9.483687283 | 3.305093795 | 12.78878108 | 245.127974 | 457.734765 | 702.86274 |
| sp\|Q08945\|SSRP1_HUMAN | 6.854658476 | 9.281812476 | 16.13647095 | 446.362085 | 29.2237162 | 475.585801 |
| sp\|Q5T8A7\|PPR26_HUMAN | 2.139737471 | 2.807064417 | 4.946801888 | 0 | 367.324615 | 367.324615 |
| sp\|P39060\|COIA1_HUMAN | 2.695496525 | 2.761702631 | 5.457199157 | 0 | 218.181634 | 218.181634 |
| sp\|P09874\|PARP1_HUMAN | 2.892396595 | 7.651580274 | 10.54397687 | 0 | 0 | 0 |
| sp\|P50914\|RL14_HUMAN | 4.095933406 | 3.305093795 | 7.401027201 | 0 | 0 | 0 |
| sp\|Q9BRU9\|UTP23_HUMAN | 2.542688948 | 4.34648648 | 6.889175427 | 0 | 0 | 0 |
| sp\|Q7Z6E9\|RBBP6_HUMAN | 2.4872289 | 4.34648648 | 6.83371538 | 0 | 0 | 0 |
| sp\|Q9UG56\|PISD_HUMAN | 4.095933406 | 2.334141337 | 6.430074743 | 0 | 0 | 0 |
| sp\|Q9HCU9\|BRMS1_HUMAN | 2.892396595 | 3.305093795 | 6.19749039 | 0 | 0 | 0 |
| sp\|Q96GK7\|FAH2A_HUMAN | 2.892396595 | 3.305093795 | 6.19749039 | 0 | 0 | 0 |
| sp\|Q96E39\|RMXL1_HUMAN | 2.542688948 | 3.305093795 | 5.847782742 | 0 | 0 | 0 |
| sp\|Q86SQ9\|DHDDS_HUMAN | 3.054540721 | 2.761702631 | 5.816243352 | 0 | 0 | 0 |
| sp\|Q9NQT5\|EXOS3_HUMAN | 2.777338118 | 2.697636338 | 5.474974456 | 0 | 0 | 0 |
| sp\|P02743\|SAMP_HUMAN | 2.892396595 | 2.542326796 | 5.434723392 | 0 | 0 | 0 |
| sp\|Q9NRC6\|SPTN5_HUMAN | 2.777338118 | 2.380354631 | 5.157692749 | 0 | 0 | 0 |
| sp\|P01036\|CYTS_HUMAN | 2.082264533 | 2.463810208 | 4.54607474 | 0 | 0 | 0 |
| sp\|Q9NWX6\|THG1_HUMAN | 2.303431313 | 2.165627548 | 4.469058862 | 0 | 0 | 0 |


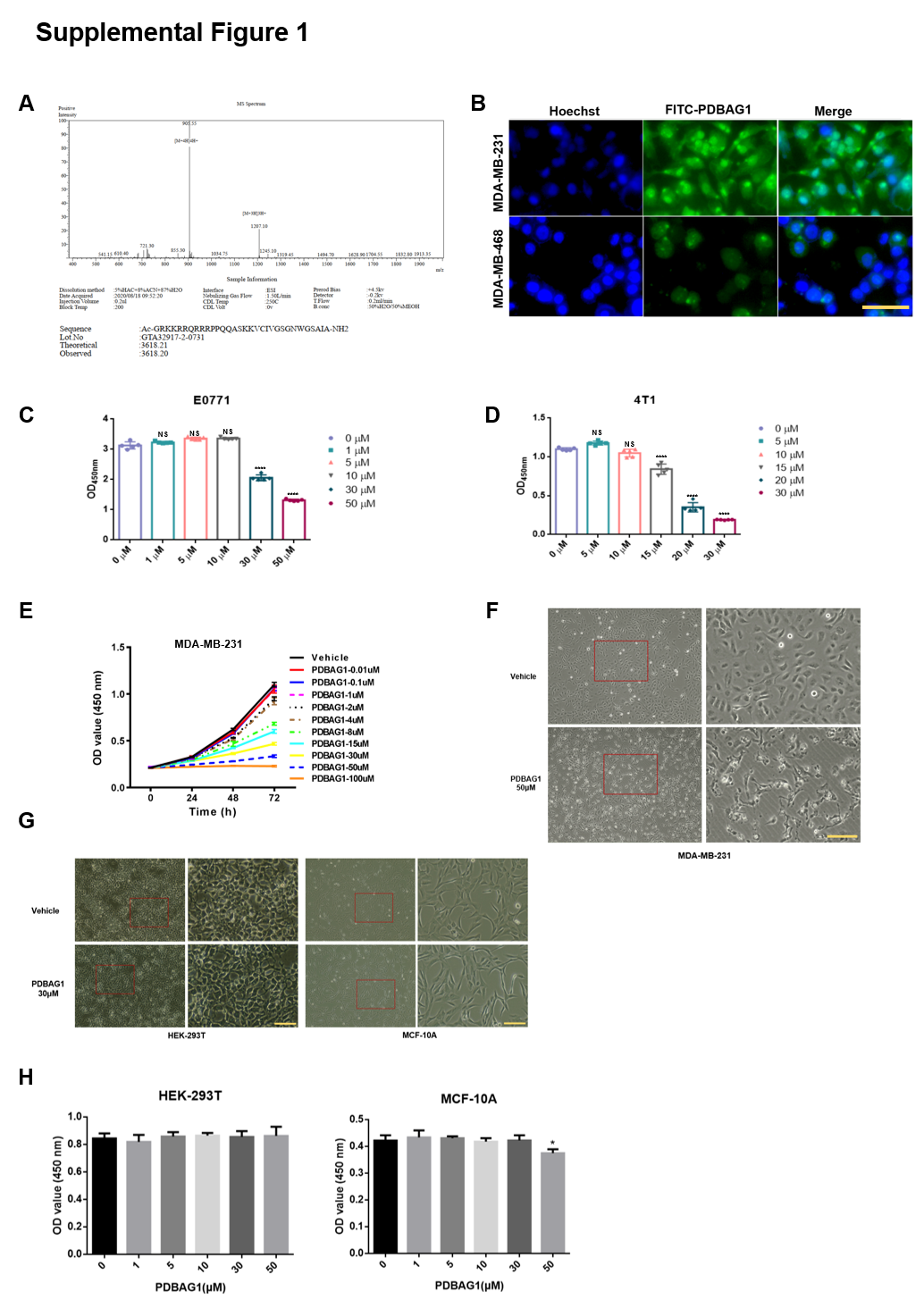


**Figure S1. PDBAG1 has the ability to permeate the cellular membrane and exert a significant inhibitory effect on various triple-negative breast cancer cell lines.**

A. The mass spectrum of PDBAG1 offers insights into its intrinsic properties, such as molecular weight and isoelectric point, providing essential characterization data.

B. Distribution of FITC-labelled PDBAG1(50 μM) after added to the cell culture for 6 hours in MDA-MB-231 and MDA-MB-468 cells. Scale bar, 50 μm.

C, D. The Cell Counting Kit-8 (CCK-8) assay revealed a pronounced inhibitory impact of PDBAG1 on the triple-negative breast cancer cell lines E0771 and 4T1, indicating a significant reduction in cellular viability.

E. OD values were detected after 24, 48 72 hours of treatment with different concentrations of PDBAG1 in MDA-MB-231 cells.

F. After 24 hours of PDBAG1(50 μM) treatment, marked enhancements in morphological alterations were observed in MDA-MB-231 cell line. Scale bar, 50 μm.

G. After 24 hours of PDBAG1(30 μM) treatment, no obvious in morphological alterations were observed in HEK-293T and MCF-10A cell lines. Scale bar, 50 μm.

H. OD values were detected after 48 hours of treatment with different concentrations of PDBAG1 in HEK-293T and MCF-10A cells(n=4). **P* < 0.05, compared to vehicle group.


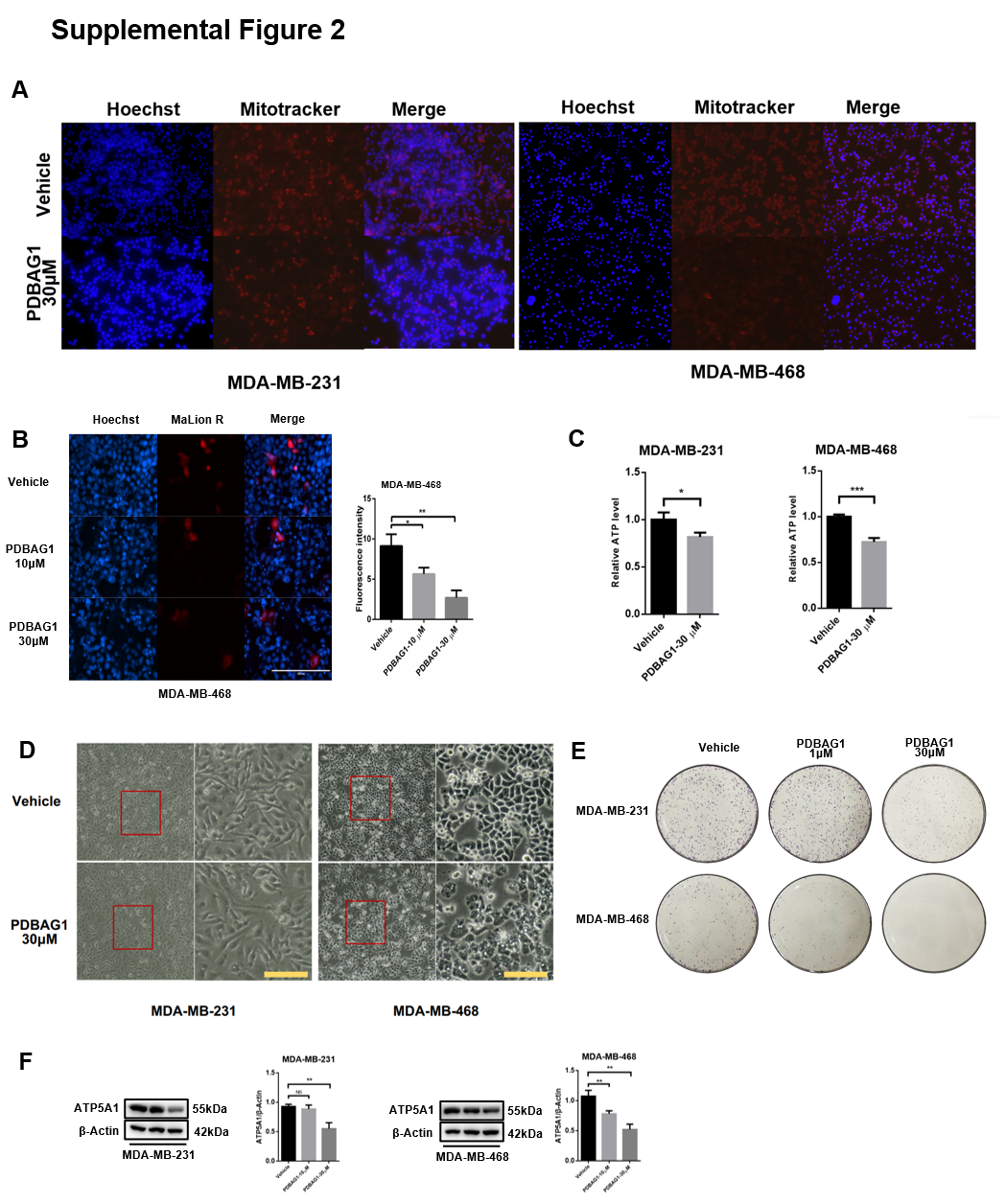


**Figure S2.** **PDBAG1 has a significant impact on the mass and morphology of mitochondria in triple negative breast cancer cells, leading to a notable inhibition of cell morphology and proliferation.**

A. Modulations in mitochondrial mass, as indicated by Mitotracker staining, following exposure to PDBAG1 for 24 hours.

B. The cellular ATP levels were measured in MDA-MB-468 cells following treatment with vehicle and PDBAG1, using the MalionR ATP sensor. Scale bar, 50 μm. A bar graph quantifies the fluorescence intensity, data are expressed as mean ± SD, with statistical significance indicated (EVOS FL AUTO, Life, ***P* < 0.01, ****P* < 0.001).

C. The cellular ATP level after vehicle and PDBAG1 treatment in TNBC cells.

D. After 24 hours of PDBAG1 treatment, marked enhancements in morphological alterations were observed in triple-negative breast cancer (TNBC) cells, specifically within the MDA-MB-468 cell line. Scale bar, 50 μm.

E. The cell clone formation assay demonstrated a pronounced inhibition of both MDA-MB-231 and MDA-MB-468 cells following treatment with PDBAG1. Notably, an increased concentration of PDBAG1 in MDA-MB-468 cells correlated with a significant reduction in the formation of tumor cell clones, indicative of a dose-dependent impact on clonogenic potential.

F. The protein expression level and relative expression statistics of ATP5A were assessed using Western blot analysis.


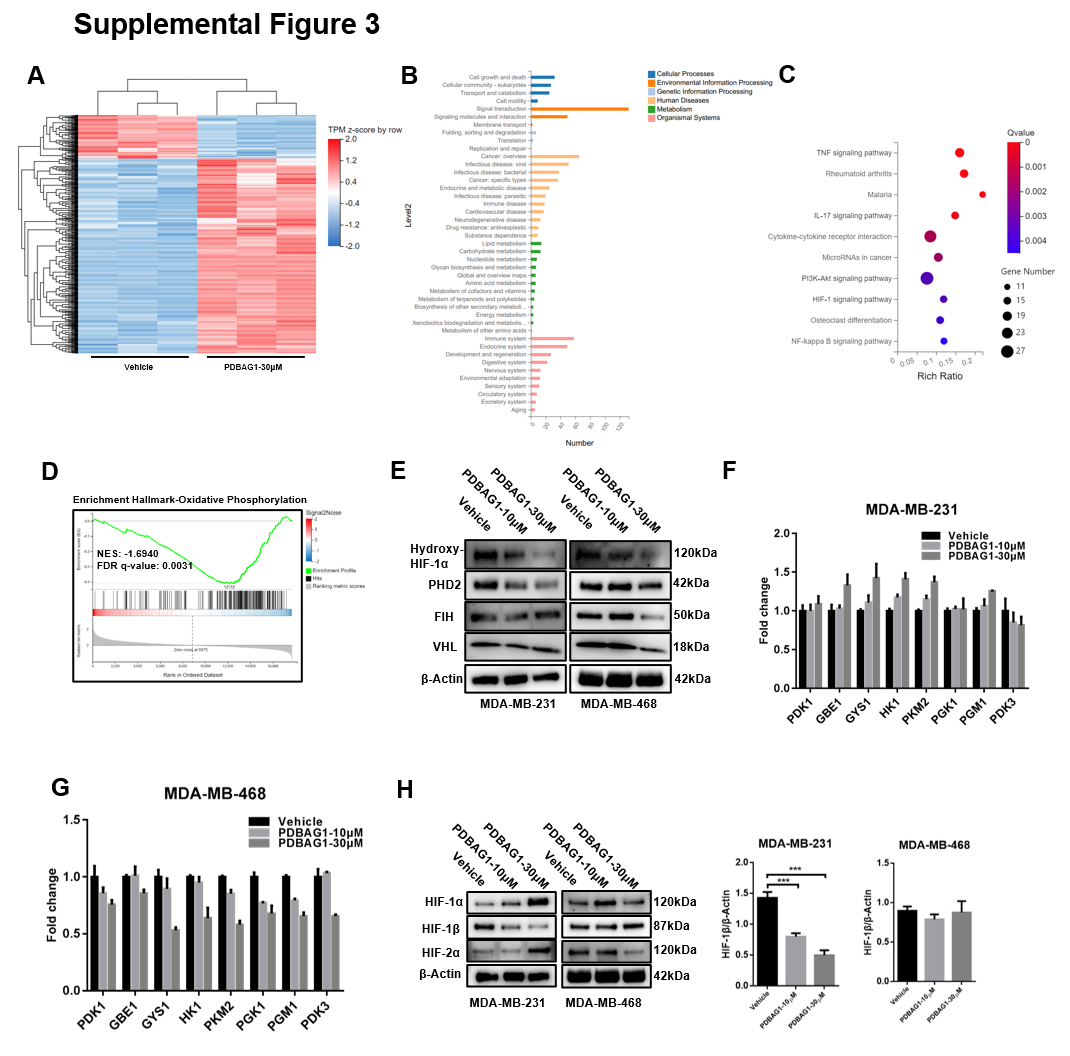


**Figure S3. PDBGA1 has significant influence on the transcriptome of triple negative breast cancer and leads to an incomplete activation of the HIF pathway.**

A, B, C. The RNA-seq was conducted subsequent to the cellular treatment with PDBAG1, and the results were summarized utilizing a heat map, GO analysis, and enrichment analysis.

D. GSEA enrichment analysis in Hallmark dataset of oxidative phosphorylation (NES=-1.6940, FDR q value=0.0031).

E. Protein levels of HIF-1α signaling pathway related proteins after treatment with different concentrations of PDBAG1 in TNBC cell lines were detected by immunoblotting.

F, G. Expression levels of glycolysis-related HIF-regulated genes after treated with PDBAG1 for 24 hours.

H. HIF-1α, HIF-1β and HIF-2α level were detected by immunoblotting after treated with different concentrations of PDBAG1in TNBC cell lines.


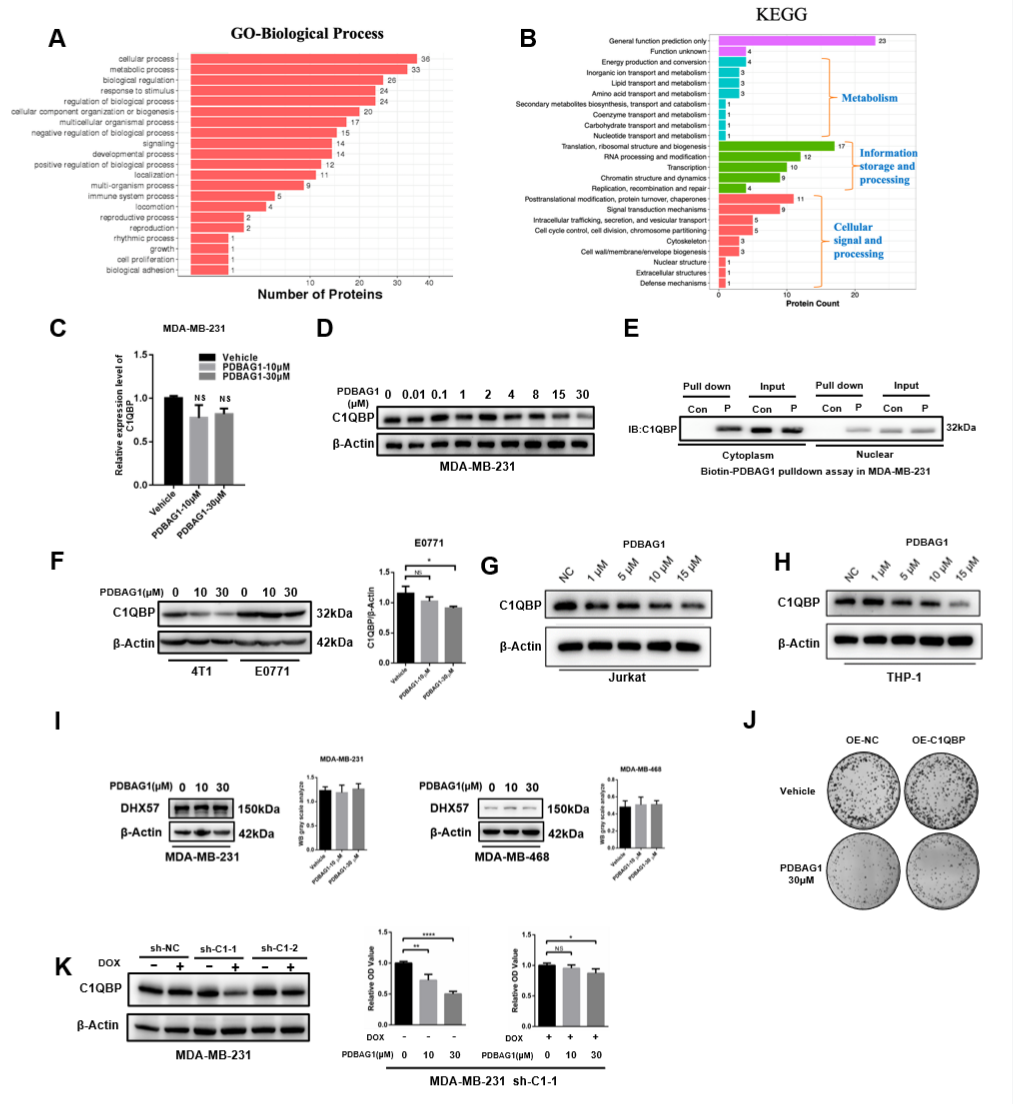


**Figure S4. Target screening and analysis of PDBAG1 confirmed its direct interaction with C1QBP, resulting in the down-regulation of C1QBP at the protein level.**

A, B. The proteins (molecular weight at about 30kDa and 150kDa) captured by PDBAG1 underwent GO and KEGG analyses as an initial functional assessment of these proteins.

C. The expression level of C1QBP mRNA was quantified using qPCR following a 24-hour treatment with PDBAG1.

D. The level of C1QBP protein was assessed in cells treated with varying concentrations of PDBAG1, revealing a concentration-dependent down-regulation trend.

E. Co-Immunoprecipitation of C1QBP with biotin-PDBAG1 by pulldown assay performed on nuclear and cytoplasmic protein samples.

F. The impact of PDBAG1 protein on the expression of C1QBP in 4T1 and E0771 triple-negative breast cancer (TNBC) cells was investigated, revealing a suppressive effect.

G, H. The Western blot analysis revealed a concentration-dependent inhibitory effect of PDBAG1 on the protein expression of C1QBP in various hematological tumor cell lines, including Jurkat and THP-1.

I. The protein level of DHX57 was assessed in cells treated with PDBAG1, remained unchanged without significant variation.

J. Stable overexpression C1QBP MDA-MB-231 cell lines were constructed to detect the clonogenicity of cells after treated with PDBAG1.

K. The inhibitory effect of PDBAG1 on cells with stable C1QBP knockdown induced by doxycycline was assessed using the CCK8 assay.


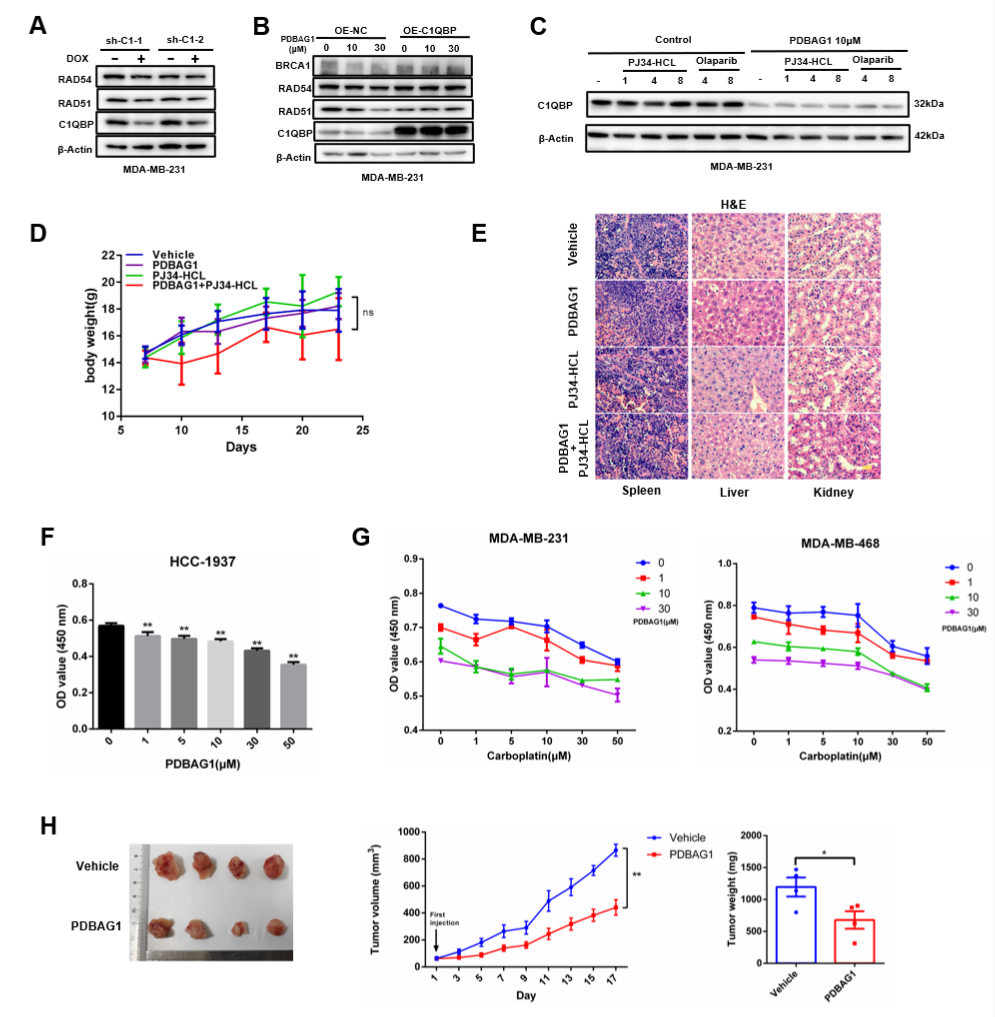


**Figure S5. C1QBP can impact proteins involved in homologous recombination repair, while PDBAG1 does not exhibit apparent toxicity in vivo.**

A. The immunoblotting analysis was employed to assess the protein expression levels of RAD51 and RAD54 following treatment with doxycycline, inducing stable knockdown of C1QBP in a triple-negative breast cancer (TNBC) cell line.

B. The protein expression levels of BRCA1, RAD51, and RAD54 were assessed through immunoblotting following treatment with PDBAG1 in a stable overexpression cell line of C1QBP.

C. The impact of PJ34-HCL and Olaparib, in combination with PDBAG1, on the protein expression levels of C1QBP was assessed using Western blot analysis following a 24-hour treatment of cells.

D. Changes in body weight of nude mice during drugs (PDBAG1 and PJ34-HCL) administration.

E. The histomorphology of liver, spleen and kidney were analyzed by H&E staining, Scale bar, 20 μm.

F. OD values were detected after 48 hours of treatment with different concentrations of PDBAG1 in HCC-1937 cells(n=4). ***P* < 0.01, compared to vehicle group.

G. TNBC cells were treated with corresponding concentrations of PDBAG1 and carbopltin for 48h in MDA-MB-231 and MDA-MB-468 cells, and the OD value of the treated cells was measured by CCK8 method.

H. Using vehicle, PDBAG1 administration in mice tumorigenic model experiments (n = 4 mice in each group), the effects of PDBAG1 on tumor image, tumor volume and tumor weight were examined (NS, non-significant, *P < 0.01, **P < 0.001, Student’s t-test. Error bars, mean ± SD, n =4).

**Peptide synthesis**

1. Weigh 3 g of RINK resin (degree of substitution 0.3 mmol/g) into a 150 ml reactor and soak it with 50 ml of dichloromethane (DCM).

2. After 2 hours, the resin was washed with nitrogen-dimethylformamide (DMF) with 3 times the resin volume, and then drained. This was repeated four times, and the resin was drained for use.

3. Add a certain amount of 20% piperidine (piperidine/DMF) to the reactor and shake it on a decolorizing shaker for 20 minutes to remove the Fmoc protecting group on the resin. After deprotection, it was washed four times with 3 times the resin volume of DMF, and then drained.

4. Take a small amount of resin and test it by ninhydrin (Ninhydrin hydrate) method (two drops of test A and test B, respectively, and react at 100 °C for 1 min). The resin is colored, indicating that the deprotection is successful.

5. Weigh an appropriate amount of the first C-terminal amino acid Ala and an appropriate amount of 1-hydroxy-benzotriazole (HOBT) into a 50ml centrifuge tube, add 20ml of DMF to dissolve it, and then add 3ml of N,N-Diisopropylcarbodiimide (DIC) was shaken for 1 min. After the solution was clear, it was added to the reactor, and then the reactor was placed in a shaker at 30 °C for reaction.

6. After 2 hours, endcap with a certain amount of acetic anhydride (acetic anhydride: DIEA: DCM = 1: 1: 2) for half an hour, then wash four times with DMF of 3 times the resin volume, and drain for use.

7. Add a certain amount of 20% piperidine (piperidine/DMF=1:4) to the reactor and shake it on a decolorizing shaker for 20 min to remove the Fmoc protecting group on the resin. After deprotection, it was washed four times with DMF and then drained.

8. Take a small amount of resin and test it by ninhydrin (Ninhydrin hydrate) method (two drops each of test A and test B, react at 100°C for 1 min), the resin is colored, indicating that the deprotection is successful.

9. Weigh an appropriate amount of the second amino acid isoleucine and an appropriate amount of HOBT into a 50 ml centrifuge tube, add 25 ml of DMF to dissolve it, then add 2.5 ml of DIC and shake for 1 min. After the solution was clarified, add the solution to the reactor and then the reactor was placed in a shaker at 30°C to react.

10. After 1 hour, take a small amount of resin for detection, and use the ninhydrin method to detect (two drops each of test A and test B, and react at 100 °C for 1 min). If the resin is colorless, the reaction is complete; if the resin is colored, it indicates condensation. Incomplete, continue to react.

11. After the reaction is complete, wash the resin four times with DMF, then drain it, add a certain amount of 20% piperidine (piperidine/DMF = 1:4) to the reactor, and shake it on a decolorizing shaker for 20 minutes to remove the residue. This removes the Fmoc protecting group on the resin. After deprotection, it was washed four times with DMF, and then dried to check whether the protection was removed.

12. Follow steps 9-11 to connect the following amino acids A, S, G, W, N, G, S, G, V, I, C, V, K, K, S, A, Q, Q, P, P, R, R, R, Q, R, R, K, K, R, G.

13. After removing the Fmoc of N-terminal Gly, endcap with a certain amount of acetic anhydride (acetic anhydride: DIEA: DCM = 1: 1: 2) for half an hour, wash with DMF four times, and then drain the resin with methanol. Then use 95 cutting solution (trifluoroacetic acid: 1, 2 ethanedithiol: 3, isopropylsilane: water = 95:2:2:1) to cut the polypeptide from the resin (add 10 ml cutting solution per gram of resin) ), and centrifuged four times with ice ether (cutting solution: ether = 1:9). Finally, it was separated and purified by HPLC, and then lyophilized to obtain a certain purity of the polypeptide.
